# Teneurin and TCAP Phylogeny and Physiology: Molecular Analysis, Immune Activity, and Transcriptomic Analysis of the Stress Response in the Sydney Rock Oyster (*Saccostrea glomerata*) Hemocytes

**DOI:** 10.3389/fendo.2022.891714

**Published:** 2022-06-17

**Authors:** Tomer Abramov, Saowaros Suwansa-ard, Patricia Mirella da Silva, Tianfang Wang, Michael Dove, Wayne O’Connor, Laura Parker, David A. Lovejoy, Scott F. Cummins, Abigail Elizur

**Affiliations:** ^1^ Centre for Bioinnovation, University of the Sunshine Coast, Sippy Downs, QLD, Australia; ^2^ School of Science, Technology and Engineering, University of the Sunshine Coast, Sippy Downs, QLD, Australia; ^3^ Invertebrate Immunology and Pathology Laboratory, Department of Molecular Biology, Federal University of Paraíba, João Pessoa, Brazil; ^4^ New South Wales (NSW) Department of Primary Industries, Port Stephens Fisheries Institute, João Pessoa, Para´ıba, Taylors Beach, NSW, Australia; ^5^ School of Biological, Earth and Environmental Sciences, University of New South Wales, Kensington, NSW, Australia; ^6^ Department of Cell and Systems Biology, University of Toronto, Toronto, ON, Canada

**Keywords:** teneurin, TCAP, stress, metabolism, ROS, phagocytosis, hemocytes, oysters

## Abstract

Teneurin C-terminal associated peptide (TCAP) is an ancient bioactive peptide that is highly conserved in metazoans. TCAP administration reduces cellular and behavioral stress in vertebrate and urochordate models. There is little information for invertebrates regarding the existence or function of a TCAP. This study used the Sydney rock oyster (SRO) as a molluscan model to characterize an invertebrate TCAP, from molecular gene analysis to its physiological effects associated with hemocyte phagocytosis. We report a single *teneurin* gene (and 4 *teneurin* splice variants), which encodes a precursor with TCAP that shares a vertebrate-like motif, and is similar to that of other molluscan classes (gastropod, cephalopod), arthropods and echinoderms. TCAP was identified in all SRO tissues using western blotting at 1-2 different molecular weights (~22 kDa and ~37kDa), supporting precursor cleavage variation. In SRO hemolymph, TCAP was spatially localized to the cytosol of hemocytes, and with particularly high density immunoreactivity in granules. Based on ‘pull-down’ assays, the SRO TCAP binds to GAPDH, suggesting that TCAP may protect cells from apoptosis under oxidative stress. Compared to sham injection, the intramuscular administration of TCAP (5 pmol) into oysters modulated their immune system by significantly reducing hemocyte phagocytosis under stress conditions (low salinity and high temperature). TCAP administration also significantly reduced hemocyte reactive oxygen species production at ambient conditions and after 48 h stress, compared to sham injection. Transcriptomic hemocyte analysis of stressed oysters administered with TCAP demonstrated significant changes in expression of genes associated with key metabolic, protective and immune functions. In summary, this study established a role for TCAP in oysters through modulation of physiological and molecular functions associated with energy conservation, stress and cellular defense.

## 1 Introduction

Organisms are continuously exposed to internal and external stressors. An imbalance of cellular stress-mitigating pathways is a known contributor to cellular damage, disease and mortality (1, 2). An organism’s ability to optimally react to cellular stress essentially depicts its resilience and survival when challenged (1, 2). Many molecular factors are associated with stress and resilience, including one of emerging interest, teneurin, because of its unique association with neural tissue and the modulation of stress behaviors *via* its peptide derivative, termed teneurin C-terminal associated peptide (TCAP) (3–5).

Teneurin is a type II transmembrane protein that is highly conserved in metazoans. In vertebrates, multiple isoforms can be found, named teneurin 1 to 4, having TCAP 1 to 4 respectively [reviewed by Meza-Aguilar and Boucard (6)]. In *Danio rerio* and other teleost fish, five teneurin paralogues exist (7). Comparative analysis in vertebrates determined that the teneurin precursors range from 2502 to 3133 amino acids [reviewed by Baumgartner and Wides (8)], contain an N-terminal intracellular domain, a single transmembrane domain and a large extracellular C-terminal domain (9). TCAP, a 40-41 amino acid long bioactive peptide, is located at the C-terminus and is cleaved from the precursor. A high degree of conservation is observed between TCAP sequences of different vertebrates (10, 11). Additionally, vertebrate TCAP-1 can be independently transcribed (*teneurin* exon 31), suggesting that TCAP has a unique and independent action from teneurins (10, 12).

Several biological activities have been observed following TCAP administration in murine models. TCAP was shown in numerous studies to modulate the stress response and behavior in rat models (5, 13, 14) and was demonstrated to enhance the growth and fasciculation of neurites in mouse hippocampal cells (15). A role in metabolism was also demonstrated, where TCAP-3 protected fish larvae from low metabolic activity during cold treatment (16), and in rats, TCAP-1 significantly increased glucose uptake in neurons through an insulin-independent manner (17). Moreover, evidence suggests that TCAP has neuroprotective activity by playing a role in cellular defense mechanisms to cope with chemical stress (18). For instance, TCAP can increase by 5-fold the viability of rat neural cells exposed to alkalotic pH and hypoxic conditions (18). Additionally, TCAP can upregulate the expression of several genes that contribute to cellular defense against oxidative stress, such as catalase, superoxide dismutase 1 (SOD1) and SOD copper chaperone, which consequently reduces the superoxide radicals, thus reducing cellular damage from reactive oxygen species (ROS) (18). These studies demonstrate that TCAP has positive effects on the biochemical and behavioral responses to stress in vertebrates, specifically in murine models.

There is a lack of understanding of TCAP’s potential stress mitigating effects for invertebrates. Teneurins have been identified in invertebrates such as *Ciona intestinalis* (Phylum Tunicata) (11), *Caenorhabditis elegans* (Phylum Nematoda) and *Drosophila melanogaster* (Phylum Arthropoda, class Insecta) [reviewed by Meza-Aguilar and Boucard (6)]. However, *Ciona intestinalis* is thus far the only invertebrate in which TCAP was studied and shown to affect its behavior, namely contractile behavior changes (11, 19). Nothing has been reported from animals representing the Phylum Mollusca, although numerous species, including oysters, are continuously exposed to an array of biotic and abiotic stressors (20–24).

Stress can be deleterious to oysters due to reactive oxygen species production, inflammation and reduced immune function (23, 25, 26) and is linked to increased bacterial load, disease outbreak, immunosuppression and ultimately, increased mortality (20, 22, 24). To mitigate stress, whether mechanical, chemical or biological, animals can shift their energy utilization to overcome the stressor by changing their behavior and metabolic adaptations (27). Several gene groups, including innate immune response, apoptosis, antioxidants and respiration, were shown to change in response to stress in *S. glomerata* (26, 28). Consequently, stress induces physiological changes that divert energy away from functions not directly required, such as growth, reproduction and various immune processes (22, 27, 29). The relationship between oyster immunity and stress is apparent, emphasizing the sensitivity of oyster hemocytes to both biotic and abiotic stressors (20, 22–24, 30).

There has been no functional investigation into the presence of TCAP or its function in molluscs nor studies investigating the effects of TCAP on immune functions. Several oyster species such as *Crassostrea gigas* and *Saccostrea glomerata* have well established genomic resources, in addition to documented research into stress response and resilience (22, 23, 25, 26, 31). Sydney Rock Oysters (SROs), in particular, encounter a plethora of potential stresses due to their intertidal estuarine niche that is exposed to changing environments, as well as pollutants and pathogens (32, 33). Thus, SROs are an appropriate model to investigate the role of TCAP and stress in an invertebrate.

This study aims to characterize TCAP in the SRO, including identifying the gene and its transcripts and potential elucidation of molecular mechanisms of mitigating stress. We report the identification of SRO TCAP and its effect on hemocyte physiology in SRO, including stress response based on *in-vitro* and *in-vivo* assays. These studies may contribute to stress response management and immune modulation not only in oysters but also in more complex metazoan species.

## 2 Materials and Methods

### 2.1 Experimental Animals

Adult SROs used in this study were obtained from Port Stephens Fisheries Institute, NSW Department of Primary Industry. The oysters were housed in a closed recirculating tank at 23°C ± 1°C and 34 ± 1 ppt and acclimated for 5-7 days before experiments. Animals were routinely fed with commercial microalgae concentrates: shellfish diet 1800^®^ and LPB (Reed Mariculture, USA).

Bivalve research does not require animal ethics approval in Queensland (Australia) under the animal ethics committee regulations, nonetheless, the animals were housed and handled with care to avoid unnecessary stress.

### 2.2 Identification of Genes Encoding Teneurin/TCAP

Teneurin sequences from vertebrate and invertebrate organisms (human, mouse, rat and *Ciona intestinalis)* were used in BLAST search against the SRO genome (NCBI access GCA003671525.1). Additionally, teneurin sequences were searched using tblastn with the transcriptome shotgun assembly (TSA) database of *S. glomerata*. After SRO *teneurin* was identified, the presence of putative isoforms was analyzed in transcriptome data of different *S. glomerata* tissues; hemocytes, gill, mantle, muscle, digestive system (accession number GGIC00000000.1), visceral ganglia (accessions number SAMN05231774, SAMN05231775) male gonad (SAMN05231772), female gonads (SAMN05231773) and heart (SAMN21892173). The TCAP region was predicted based on known TCAP motifs and by multiple sequence alignment, as described in Colacci, De Almeida (11). TCAP sequence alignments were generated using ClustalW, highlighting >50% sequence similarity. Similarly to the SRO identification, known teneurin sequences were searched in several species of invertebrates, then a phylogenetic tree was constructed using the Maximum Likelihood NNI method with 1000 bootstrap replications. Protease cleavage site prediction of SRO teneurin was analyzed by PROSPER (https://prosper.erc.monash.edu.au). The SRO TCAP (sroTCAP) structure was predicted using I-TASSER (34) and aligned to the crystal structure of human teneurin-2 protein (PDB 6CMX) using Chimera software (35).

### 2.3 Analysis of *Teneurin* Expression in SRO

Transcriptome data for hemocytes, gill, mantle, digestive system, male and female gonad (gonads were a mix of maturation stages), visceral ganglia and heart were available from previous studies (28, 36).

Expression levels of *teneurin* were analyzed using the CLC Genomics Workbench software version 21 (Qiagen). Raw transcriptome data were mapped against the *S. glomerata* genome, then transcript reads were normalized and transformed to RPKM (reads per kilobase million) and converted to Z-score. Relative expression of target genes was then retrieved and evaluated using a heat map generated by the ClustVis server (37).

### 2.4 TCAP Immuno-Detection

#### 2.4.1 Antibody Production

Polyclonal antibodies against synthetic sroTCAP sequence (NEILTHGSARGYEGRYRQSQTPTEYPELSDDCNSIKLQKTNR), synthesized by Royobiotech (Shanghai, China), were produced in rabbits and antibodies of 3^rd^ immunization serum were purified using a protein A column (Walter and Eliza Hall Institute of Medical Research, Australia). Serum titration showing sroTCAP specificity using ELISA is shown in **Supplementary Figure S1**.

#### 2.4.2 Western Blot Analysis

The presence of sroTCAP and its distribution in different SRO tissues was evaluated using western blots. Freshly harvested SRO tissues, 100-200 mg, (visceral ganglia, female and male gonads, sensory tentacles, mantle, gill, hepatopancreas, hind-gut, and heart) were collected from 3 animals (un-verified sexes) and placed in radioimmunoprecipitation assay (RIPA) buffer (Thermo Scientific, USA) containing proteinase inhibitor cocktail (Promega). Then, tissues were homogenized and sonicated for 5 min on ice. Cell debris was removed by centrifugation at 17,000 x g at 4°C for 15 min.

For hemolymph and hemocyte sample preparation, 500 µL of freshly extracted hemolymph was obtained from the adductor muscle of 3 animals using a 23G needle and centrifuged at 500 x g for 10 min at 4°C. The hemocyte pellet and cell-free hemolymph supernatant were separated. The hemocyte pellet was extracted as described above, while a proteinase inhibitor cocktail (Promega G6521, Promega, CA, USA) was added to the cell-free hemolymph and dried in a vacuum centrifuge (Invitrogen). The dry protein pellet was resuspended in RIPA buffer.

Protein lysates were quantified using a BCA kit (Thermo Scientific, USA). For each tissue, 30 µg total soluble proteins and 30 ng synthetic sroTCAP were boiled in Laemmli buffer (BioRad Laboratories, Hercules, CA, USA) with 5% β-mercaptoethanol for 10 min, before resolving on a 4–15% Mini-PROTEAN^®^TGX™ Precast Protein Gel (Bio-rad). Proteins were transferred onto Trans-Blot Turbo transfer pack mini nitrocellulose membrane 0.22 µm (Bio-Rad) using a Trans-blot Turbo transfer system (Bio-rad). After washing the membrane three times with tris-buffered saline containing 1% tween-20 (TBS-T), blocking buffer (3% BSA in TBS-T) was applied for 1 h at room temperature with shaking. The membrane was washed three times before incubating with 10 mL primary rabbit anti-sroTCAP (1:1000) in blocking buffer overnight at 4°C with gentle shaking. The membrane was washed three times and incubated with secondary goat anti-rabbit IR680 nm (Li-Cor) at 1:5000 dilution for 1 h at room temperature with gentle shaking. After washing three times with TBS-T, the membrane was blot dried and scanned using 700 nm wavelength on the Odyssey CLx (Li-Cor) and visualized with image studio 4.0 (Li-Cor).

#### 2.4.3 TCAP Hemocyte Localization Using Immunocytochemistry

Hemolymph was extracted from oysters *via* the adductor muscle using a 23G syringe and incubated on a glass slide (poly-L-lysine coated) for 15 min to allow cells to adhere. The cells were then fixed with 4% formalin in filter sterilized seawater (FSSW) for 10-15 min followed by 1x phosphate-buffered saline (PBS) washes and membrane permeabilization with 0.5% Triton X-100 in PBS for 10 min. Cells were blocked with 3% bovine serum albumin (BSA) in PBS for 1 h with gentle shaking. Primary anti-sroTCAP (1:1000) in PBS with 1% BSA was added and incubated overnight at 4°C with gentle shaking. Cells were incubated with pre-immune serum (1:1000) in blocking buffer in the negative control slides. After washing three times with PBS, secondary goat anti-rabbit antibodies (Alexa Fluor 488; 1:2000) were added and incubated at room temperature for 1 h with gentle shaking. After three washes in PBS, DAPI (Sigma) (1:1000) in PBS was added to stain the nuclei. Images were obtained using a Leica DM5500 microscope and Leica DFC550 camera (Leica Microsystems).

### 2.5 Pull-Down Assay: Analysis of sroTCAP Protein-Protein Interaction With SRO Total Protein Lysate

To identify candidate proteins that interact with TCAP, a pull-down assay was performed. For this, biotinylated sroTCAP was produced using a 20-fold molar excess of EZ-Link™ Sulfo-NHS-SS-Biotin, according to the manufacturer’s protocol (ThermoFisher Scientific, USA). Non-reacted biotinylation reagent was removed using a 2 K molecular weight cut-off dialysis membrane (Slide-A-Lyzer™ G2 Dialysis Cassettes; ThermoFisher Scientific, USA) in PBS, according to manufacturer’s protocol. Hemocyte pellets were collected from 3 animals as mentioned in section 2.4.2 and then lyzed in Pierce™ IP Lysis Buffer (ThermoFisher Scientific, USA) with protease inhibitor cocktail (Promega G6521, Promega, CA, USA) and homogenized on ice using a tissue homogenizer, followed by sonication bath on ice for 5 min. The homogenate was centrifuged at 12,500 x g for 15 min at 4°C, then the supernatant was transferred to a new tube and used in the pull-down assay.

Pull-down assays of biotinylated-sroTCAP with interacting proteins were performed as per manufacturer’s instructions (Pierce™ Biotinylated protein interaction pull-down kit; ThermoFisher Scientific, USA) with a few modifications. Briefly, 100 µL immobilized Streptavidin gel slurry was placed in a spin column and washed three times with Tris-buffered saline (TBS). Bait protein (sroTCAP 100 µg in 250 µL TBS buffer) was bound to the gel by incubating for 1 h at 4°C with gentle shaking. Then, the gel was washed with TBS and blocked with biotin solution. A hundred microliters of crude protein lysate containing 250 µg total soluble proteins (prey) were diluted in 200 µL TBS and incubated with beads for 2 h at 4°C with gentle shaking. Unbound proteins were rinsed with wash buffer; either weak wash (TBS) or medium stringency wash (acetate pH 5.0, NaCl 0.012 M) three times. Interacting proteins were recovered by incubating the gel beads with 200 µL elution buffer (pH 2.8) for 5 min, and the solution was neutralized with 10 µL 1M Tris solution and elution was repeated once more. Negative control pull-down was performed similarly but omitted the biotinylated TCAP binding to the gel beads. Proteins detected in the negative control were considered background binding and were deducted from the results.

Pull-down eluent was dried in a Savant SC250EXP vacuum centrifuge (Thermo Fisher), resuspended in Laemmli buffer with 5% β-mercaptoethanol and boiled for 10 min. Proteins were separated on 4–15% Mini-PROTEAN^®^TGX™ Precast Protein Gels and stained with Coomassie brilliant blue G-250. Visible bands were excised for trypsin digest. If no bands were visible, the whole lane was processed. In-gel trypsin digestion, uHPLC tandem QTOF MS/MS analyses and peptide alignment to the SRO protein database were performed as reported previously (38).

### 2.6 Effect of sroTCAP on Hemocyte Phagocytosis

Prior to assay, the oysters shell edge was clipped using a bone cutter to expose the inner cavity, and then oysters were allowed to recover for two days. The gap created access for injections (using a 27 G needle) and hemolymph withdrawal (using a 23 G needle).

#### 2.6.1 Evaluation of sroTCAP Minimum Effective Dose at Ambient Conditions

Firstly, a dose-response assay was performed to evaluate the effective dose of sroTCAP that affects phagocytosis activity in SRO. The minimum sroTCAP concentration used was established based on rat studies (3), where 1 pmol was injected per 1 gram of animal’s weight. The average flesh weight of SRO was ~5 g; hence, 5 pmol per animal was a starting point. Five animals per treatment were injected intramuscularly (IM) with 0 (control), 5, 10 and 20 pmol TCAP in 50 µL FSSW. Following injections, the oysters were left outside the water for 10 min then placed in a 10 L container filled with 4 L of seawater (34 ppt) at ambient temperature (22°C) for 3 h. Hemolymph was then extracted and used for phagocytosis assay performed as described below in “Phagocytosis plate assay”.

#### 2.6.2 Effect of TCAP on Phagocytosis in Stress Challenged Oysters

Oysters were injected with 5pmol sroTCAP in 50 µL FSSW (n=7) or 50µL FSSW as a control (n=5). The stress treatments were divided into three time points: 3, 12 and 24 h under stress conditions and ambient controls were treated for 24 h. Following injections, the oysters were incubated outside the water for 10 min then placed in 10 L tub containing 4 L of water; The conditions in the stress treated groups consisted of low salinity (15 ppt) and high temperature (30 ± 1°C), while the ambient control (non-stress group) were exposed to 34 ppt and 22 ± 1°C [stress conditions modified from Ertl and O’Connor (26)]. Each tank setup included a water pump to circulate the water for gas exchange and a water heater to maintain the desired water temperature. Following incubation at the different conditions, the hemolymph was extracted, and an *ex-vivo* phagocytosis plate assay was performed as described below.

#### 2.6.3 Phagocytosis Plate Assay

Immediately after the experimental treatments and exposures were completed, hemolymph was withdrawn. As much cavity water as possible was removed before hemolymph sampling by placing the oyster cut side down on an absorbent paper for several minutes until the animals were processed. Hemolymph was extracted from the adductor muscle sinus and kept on ice to limit aggregation and adhesion. Hemolymph from each animal was observed under the microscope for contamination, and samples containing noticeable bacteria or protozoa were discarded. Then, the hemolymph was passed through an 80 µm mesh to eliminate potential tissue fragments and aggregates.

Fluorescent latex beads (L-4530, Sigma) were prepared by diluting 30 µL beads slurry in 1 mL total FSSW followed by strong vortexing. It was observed that beads tended to flocculate when stored, causing outliers, and those were removed. Therefore, before each assay, the bead solution was sonicated in a bath for 5 min to facilitate proper bead dispersal.

The plate assay was performed in a Nunclon delta surface 96-well plate (Thermo scientific) and kept on ice during hemolymph dispensing. After 100 µL hemolymph samples were dispensed (in triplicates), the plate was removed from the ice and allowed to incubate at ambient temperature for 15 min to allow cells to adhere to the plate surface. Then, 10 µL of latex bead working solution was added to each sample well and dispersed by gently tapping the plate on all sides. The plate was incubated at ambient temperature for 40 min in the dark, followed by aspiration to wash the unbound beads. Then, 100 µL FSSW was gently added to each well and aspirated as above. The cells were then fixed (5 min) and washed with 4% formalin in FSSW, followed by the addition of 100 µL of 4% formalin in FSSW to each well before reading the plate at λ_ex_ 470 nm; λ_em_ 505 nm (EnSpire^®^ Multimode Plate Reader).

### 2.7 Effect of sroTCAP on Hemocyte ROS Production in Stress-Challenged Oysters

ROS production was evaluated using 2’,7’-dichlorodihydrofluorescein diacetate (H_2_DCFDA) (Sigma D6883). This non-fluorescent, membrane-permeable probe is oxidized in the presence of ROS to a highly fluorescent compound, 2’,7’-dichlorofluorescein (DFC). Therefore, the number of cells that emit fluorescence is directly proportional to ROS levels (39).

Oysters (n=6) were injected with either 5 pmol sroTCAP or FSSW (as mentioned in section 2.6.2), before being subjected to (1) Ambient control – 35 ppt and 22 ± 1°C for 24 h or (2) Stress – 15 ppt and 30 ± 1°C for 12, 36 and 48 h. After each treatment, hemolymph was withdrawn (as described in section 2.6.3), and 100 µL were transferred to a Nunclon delta surface 96-well plate (Thermo scientific) in duplicate. Cells were allowed to adhere to the plate at room temperature for 15 min. Then, 1 µL of 0.5 mM H_2_DCFDA was added to each well to provide a final concentration of 5 µM. The plate was then incubated in the dark for 1 h at room temperature, prior to fluorescence reading at λ_ex_ 490 nm; λ_em_ 520 nm (EnSpire^®^ Multimode Plate Reader).

#### 2.7.1 Evaluation of the Potential Cytotoxic Effects of sroTCAP on Oyster Hemocytes

Hemolymph from four adult SROs (unverified sex) was withdrawn, processed and dispensed in triplicates into a 96-well plate as mentioned in section 2.6.3. Following hemocyte adherence, the supernatant (hemolymph fluid) was aspirated replaced with 100 µL of propidium iodide (PI) solution (50 µg/mL in FSSW) and the background fluorescence was read at λ_ex_ 544 nm; λ_em_ 612 nm (EnSpire^®^ Multimode Plate Reader). Then, the PI solution was removed and replaced with 100 µL PI solution containing 0.1, 1, 10, 100 and 1000 pmol/mL of sroTCAP. Hydrogen peroxide (8 µM and 80 µM in PI solution) was used as a positive control. The plate was incubated at ambient temperature in the dark for 180 min, and fluorescence was measured. Then, to acquire 100% mortality reading, 2 µL Triton-X was added and gently agitated in an orbital shaker for 15 min to lyze all cells before max fluorescence was read. The percent death (cytotoxicity %) was calculated with the equation below, where background = initial fluorescence of cells with PI solution, sample OD = measured fluorescence at 180 min post-treatment, and Max= fluorescence after complete cell lysis.


$$Cytotoxicity\;\%\;=\;\frac{Sample\;OD\;-\;Background}{Max\;-\;Background}\times \;100$$

### 2.8 Transcriptome Analysis of Hemocytes in Stressed Oysters Following TCAP Injection

In this experiment, only adult female SRO were used to limit sex-related variation. Four groups of SRO (n=6 each) were used: (1) injected with sroTCAP (5 pmol in 50 µL FSSW) exposed to stress, named ST. (2) Injected with 50 µL FSSW and exposed to stress, named SS. (3) Injected with sroTCAP (5 pmol in 50 µL FSSW) exposed to ambient, named AT, and (4) injected with 50 µL FSSW and exposed to ambient named AS. Following injections, oysters were incubated outside the water for 10 min to allow the treatment to circulate in the hemolymph. Then, oysters were placed into 4 L of water for 3 h under ambient or stress conditions (**Table 1**).

**Table 1:** Summary of injection and incubation conditions used for hemocyte transcriptome analysis.

Treatment	Incubation condition (n=6 each)	Aim
5 pmol sroTCAP	Ambient (34 ppt and 22 ± 1°C)	Determine TCAP effect at ambient
5 pmol sroTCAP	Stress (low salinity 15 ppt and high temperature 30 ± 1°C)	Determine TCAP effect under stress
FSSW	Ambient (34 ppt and 22 ± 1°C)	Control at ambient
FSSW	Stress (low salinity 15 ppt and high temperature 30 ± 1°C)	Control under stress

At the end of the treatments, hemolymph was collected as described in section 2.6.3 and hemocytes were separated as described in section 2.4.2. Two hemocyte pellet samples per condition were pooled, then snap-frozen in liquid nitrogen and stored at -80°C. Total RNA extraction was performed using TRIzol reagent (ThermoFisher) according to the manufacturer’s protocol. Non-directional poly A library was prepared using NEBNext^®^ Ultra RNA Library Prep Kit for Illumina^®^ and sequenced using NovaSeq 6000 PE150 by Novogene (Hong Kong) and generated over 18 million reads (~ 6 GB). Raw reads were deposited in the NCBI SRA database under accession PRJNA804582. Raw transcriptome data processing; QC check, trimming, mapping, and pair-wise differential gene expression (DGE) was done using CLC genomic version 21 (Qiagen) using default parameters except for mapping in which a length fraction of 0.5 and similarity fraction of 0.8 was used.

DGE of each pair-wise comparison list was filtered with a False Discovery Rate (FDR) value of ≤0.05 and log2 fold change of > 1 and < -1. Filtered DGE from each comparison was used to obtain the enriched gene ontology (GO) terms using Omics-box (BioBam, Spain 2020) with default parameters. Then, REVIGO was used to obtain the most representative GO terms from the enrichment list (40). A dispensability cut-off of <0.1 was used to select the most representative GO terms. Then, to visualize the enriched GO terms after filtering, the GO name, gene number, log size, and P-value were used in RStudio as previously described (41).

A list of genes associated with immune-related genes, antioxidants, apoptosis, respiration, heat shock proteins (HSPs), and osmoregulation in oysters were obtained based on literature (26, 28) and searched in the filtered list of DGE in all the treatment comparisons. Then, a full list of genes of interest and their corresponding transcript Counts Per Million (CPM) from all the treatments and replicates were converted to log2 and were used to generate a heatmap to visualize the expression patterns among the groups using the ClustVis server (37). Dendrogram clustering distance of rows and columns were based on correlation using average as method.

### 2.9 Statistical Analysis

All results are expressed as mean ± SEM. Statistical significance of phagocytosis experiments and cytotoxicity assay was assessed by one-way ANOVA, Fisher’s least significant difference (LSD) and paired t-test for the ROS. A *P*-value < 0.05 was considered statistically significant.

## 3 Results

### 3.1 Characterization of Teneurin in the Sydney Rock Oyster, *S. glomerata*

**Figure 1:**
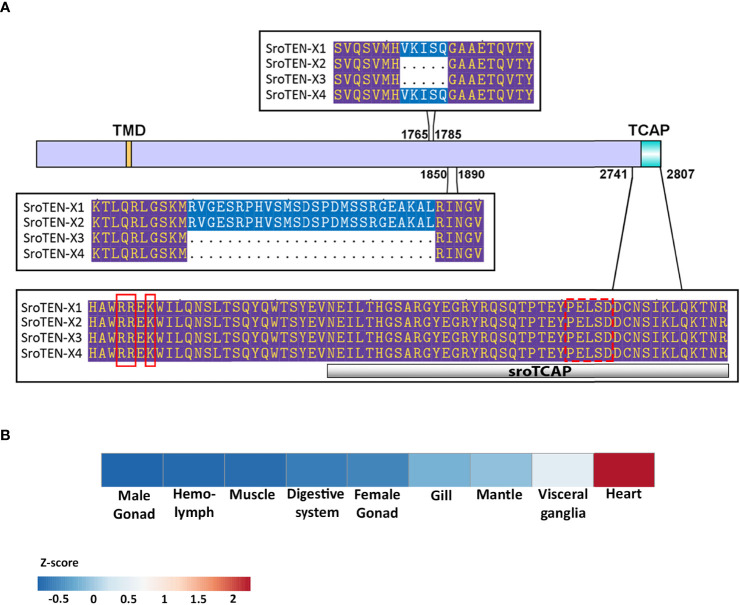
Teneurin predicted amino acid sequence and gene expression in SRO. **(A)** *S. glomerata* teneurin splice variants; blue shading represented regions that were present in two out of the four isoforms. Purple shading indicates 100% sequence similarity. TMD- a single transmembrane domain. The amino acid sequence of the synthesized sroTCAP peptide (42 aa) used in this study is shown as a grey bar. Solid red boxes depict predicted cleavage sites (leading to 60 aa TCAP), and the dotted red box shows the conserved PELSD motif. **(B)** Heatmap of teneurin expression in different SRO tissues, normalized with Z-score analysis.

*In silico* search of the *S. glomerata* genome led to the identification of a single *teneurin* gene, while transcriptome data revealed the presence of 4 different splice variants in which the predicted TCAP region was conserved (**Figure 1A**). Full sequences of teneurin splice variants are presented in **Supplementary Table S1**. The predicted SRO teneurin protein isoforms ranged between 2776 to 2807 amino acid residues or 313.63 kDa to 316.96 kDa. Predicted proteolytic cleavage sites upstream of the TCAP region (sroTeneurin isoform x1, amino acid position 2743-2744) suggest that TCAP is liberated from teneurin post-translationally, resulting in a 60 amino acid TCAP (7.09 kDa). All four isoforms of SRO TCAP possess the conserved P-E-L-S-D sequence, in line with the reported P/L-E-L-A/S/T-D sequence, which is common to all vertebrate TCAPs (11). However, it lacks the glycine residue and the dibasic cleavage site at the C-terminus, thus signifying no C-terminal amidation. SRO *teneurin* gene expression was observed across all tissues analyzed, with the highest expression in the heart, followed by the visceral ganglia (**Figure 1B**).

**Figure 2:**
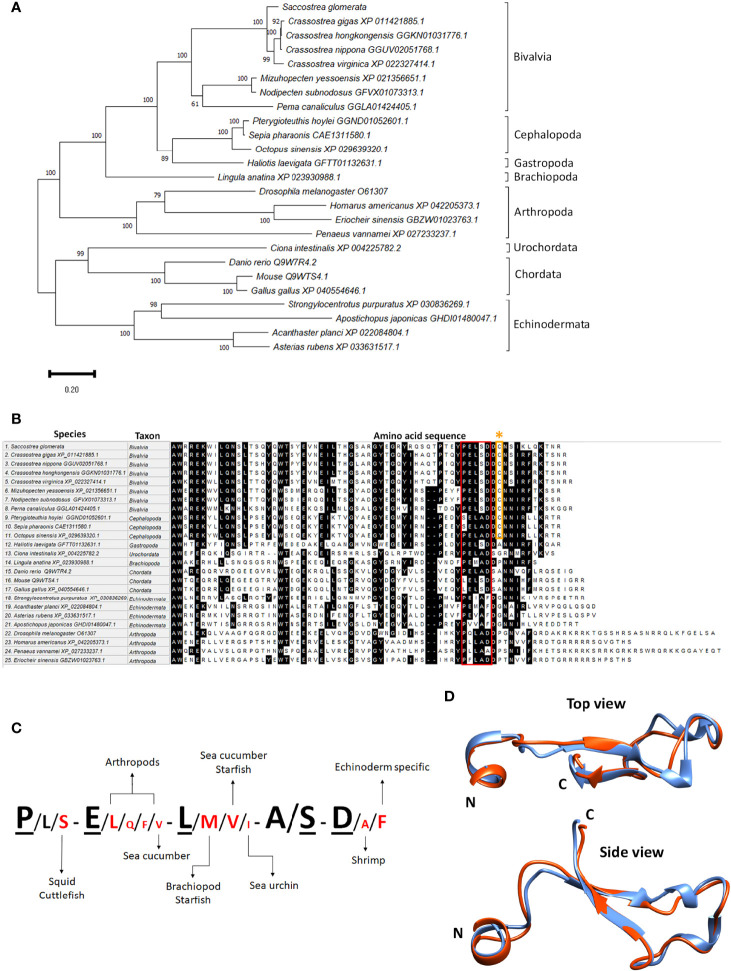
Teneurin/TCAP sequence analysis. **(A)** Phylogenetic tree of various invertebrates and vertebrates showing relationship based on teneurins, analyzed using Maximum Likelihood, 1000 bootstrap.**(B)** Homology alignment of TCAP sequences from invertebrates and vertebrates using MUSCLE, highlighting amino acid residues where >50% homology is observed. The red box shows the conserved motif among invertebrates and vertebrates. The orange asterisk shows the conserved cysteine residues among bivalvia and chepholopoda. **(C)** Conserved TCAP residues in various species show species specificity. Underlined residues- SRO and bivalve specific, red residues- newly identified in molluscs, echinoderms and arthropods. The size of residues reflects the relative level of conservation between the species investigated. The larger the size, the higher frequency a specific residue appears in the dataset and vice versa. **(D)** 3D model alignment of predicted sroTCAP with human TCAP-2, showing a top and side view. Blue ribbon- sroTCAP, red ribbon- human TCAP-2. N and C depict the N- and C- terminus, respectively.

Teneurin sequences of several species in different phyla were identified, including cephalopods and crustaceans, where a single *teneurin* gene was found, having multiple isoforms. Phylogenetic analysis of the full-length teneurin from multiple species showed that despite the conserved motifs, teneurins of closely related organisms cluster together in separate clades (**Figure 2A**). A comparative species TCAP sequence alignment showed that it has highly conserved regions between vertebrates and invertebrates (**Figure 2B**). Only vertebrate and two crustacean TCAPs contain a glycine residue followed by a dibasic cleavage site K/R-K/R at the C-terminus, which is predicted to be post-translationally processed, leading to amidation (42). This motif is absent from the other invertebrate TCAPs, and therefore the C-terminal TCAP is expected to contain an –OH group. SRO and other bivalve TCAP’s examined contain a conserved P-E-L-S-D domain (**Figures 1A** **,** **2B**). Most of the unique residues within the conserved domain are present mainly in echinoderms and arthropods (**Figure 2C**). Bivalve and cephalopod TCAPs contain a conserved cysteine residue, which is absent in vertebrate and other invertebrate TCAPs analyzed. The human teneurin-2 3D structure (determined by x-ray crystallography) was aligned and its TCAP region was compared with the sroTCAP predicted structure and showed high similarity (**Figure 2D**).

### 3.2 Detection of the TCAP Peptide in SRO and Spatial Localization in Hemocytes

**Figure 3:**
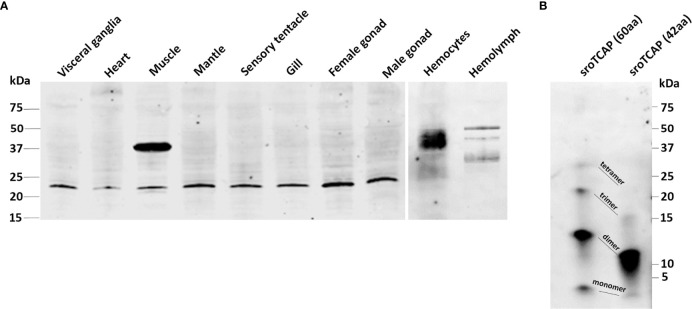
Western blot immuno-detection of TCAP in SRO tissues. Western blot using **(A)** 30 μg of total soluble protein from each tissue, and **(B)** 30 ng of 60 and 42 amino acid **(aa)** synthetic sroTCAP.

An antibody specific to sroTCAP was generated and used to identify several immunoreactive (ir) protein bands in SRO tissues based on western blots (**Figure 3A**). Bands generally corresponded to the size of cleaved variants of teneurin (with TCAP still attached) but with a single predominant ~22 kDa band detected in most tissues (except hemolymph). Muscle, hemocytes and hemolymph were the only tissues with higher molecular weight ir-TCAP bands of ~37 kDa. No ir protein was observed at ~7.09 kDa, the predicted molecular weight of the cleaved sroTCAP. Synthetic sroTCAP peptides (42 and 60 residues) were observed at molecular weights corresponding to monomers and multimers (**Figure 3B**).

**Figure 4:**
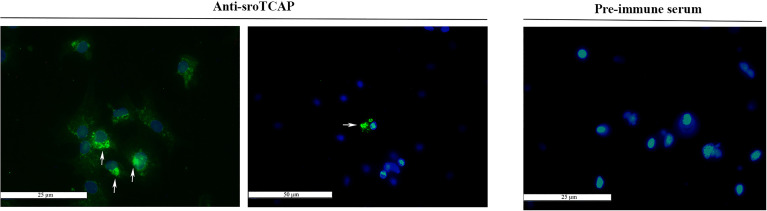
Localization of TCAP in SRO hemocytes. Left and middle: TCAP detected in the cytosol of hemocytes and in granule-like bodies (white arrows) showing 25 and 50 µm scale bar respectively. Right: No florescence was observed when pre-immune serum was used, scale bar 25 µm. Green signal: ir-TCAP. Blue signal: DAPI stained nuclei.

The presence of sroTCAP in hemocytes suggested a potential role in immune functioning. Oyster hemolymph comprises two main cell types; granulocytes and agranulocytes (also called hyalinocytes). Immunofluorescent localization, using the anti-sroTCAP identified ir-protein in the cytosol of hemocytes (**Figure 4**). In some hemocytes, TCAP was localized inside organelles or granules with more intense staining than the cytosol (**Figure 4** **- arrows**). No signal was detected on the membrane surface of hemocytes (**Figure 4**). Negative control using pre-immune serum shows no ir-TCAP signal in hemocytes.

Using crude proteins derived from SRO hemocytes and biotinylated sroTCAP, Pull-down assays identified 27 unique proteins at low stringency wash, including fasciclin, monocarboxylate transporter 13-like and X-box binding protein (**Supplementary Table S2**). At medium stringency wash, two proteins were identified: extracellular superoxide dismutase (EcSOD) and glyceraldehyde-3-phosphate dehydrogenase (GAPDH).

### 3.3 Effect of TCAP on Hemocyte Phagocytosis and ROS Production

**Figure 5:**
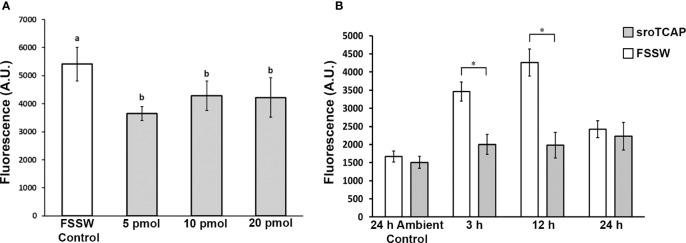
Phagocytosis activity of SRO hemocytes exposed to ambient and stress conditions injected with sroTCAP or FSSW. **(A)** Hemocyte phagocytosis activity following 3 h sroTCAP treatments (5, 10 and 20 pmol), compared to FSSW control (0 pmol). Oysters used were in ambient conditions of 34 ppt and 22°C (non-stressed). Lowercase letters indicate significant differences between treatments (ANOVA, p < 0.05). **(B)** Hemocyte phagocytosis activity at different time points (3, 12 and 24 h) post-stress (30°C, 15 ppt) following IM administration of 5 pmol sroTCAP or FSSW (control) treatment. Ambient control (non-stressed 34 ppt and 22°C) shows hemocyte phagocytosis activity 24 h post 5 pmol sroTCAP or FSSW IM administration. Y-axis shows the arbitrary fluorescence units, which reflects the phagocytosis activity; higher fluorescence demonstrates higher activity and vice versa (*P < 0.001).

To determine whether TCAP affects SRO hemocyte phagocytosis activity, and at what minimal effective dose, sroTCAP (5-20 pmol) was injected into non-stressed oysters, and phagocytosis activity was assessed. Phagocytic activity was significantly reduced at all peptide concentrations compared to no peptide controls (**Figure 5A**), and no cytotoxic activity was observed at concentrations from 0.1 to 1000 pmol/mL, where the maximum dose was 1000-fold higher than that used for sroTCAP injections (**Supplementary Figure S2**). Therefore, 5 pmol sroTCAP was used in the subsequent experiments to assess the effects of TCAP on hemocyte phagocytosis when SROs were stress-challenged (low salinity, high temperature).

Oysters kept under stress conditions showed significantly higher hemocyte phagocytosis activity than non-stressed (ambient condition) individuals at 3 h and peaked after 12 h of stress (*P* < 0.001) (**Figure 5B**). After 24 h of stress, oyster hemocyte phagocytic activity declined and was not significantly different to ambient control. Under the same stress conditions, a single dose of 5 pmol sroTCAP significantly reduced hemocyte phagocytosis activity at 3 and 12 h of stress, compared to the FSSW control group (*P* < 0.001). Furthermore, the peptide-administered groups were not significantly different to each other at different stress durations and remained at a baseline level. Overall, the results showed that when sroTCAP was administered to oysters, phagocytosis activity under stress was maintained at a level similar to that of non-stressed individuals. Qualitative images of hemocyte phagocytosis of fluorescent latex beads are shown in **Supplementary Figure S3**.

**Figure 6:**
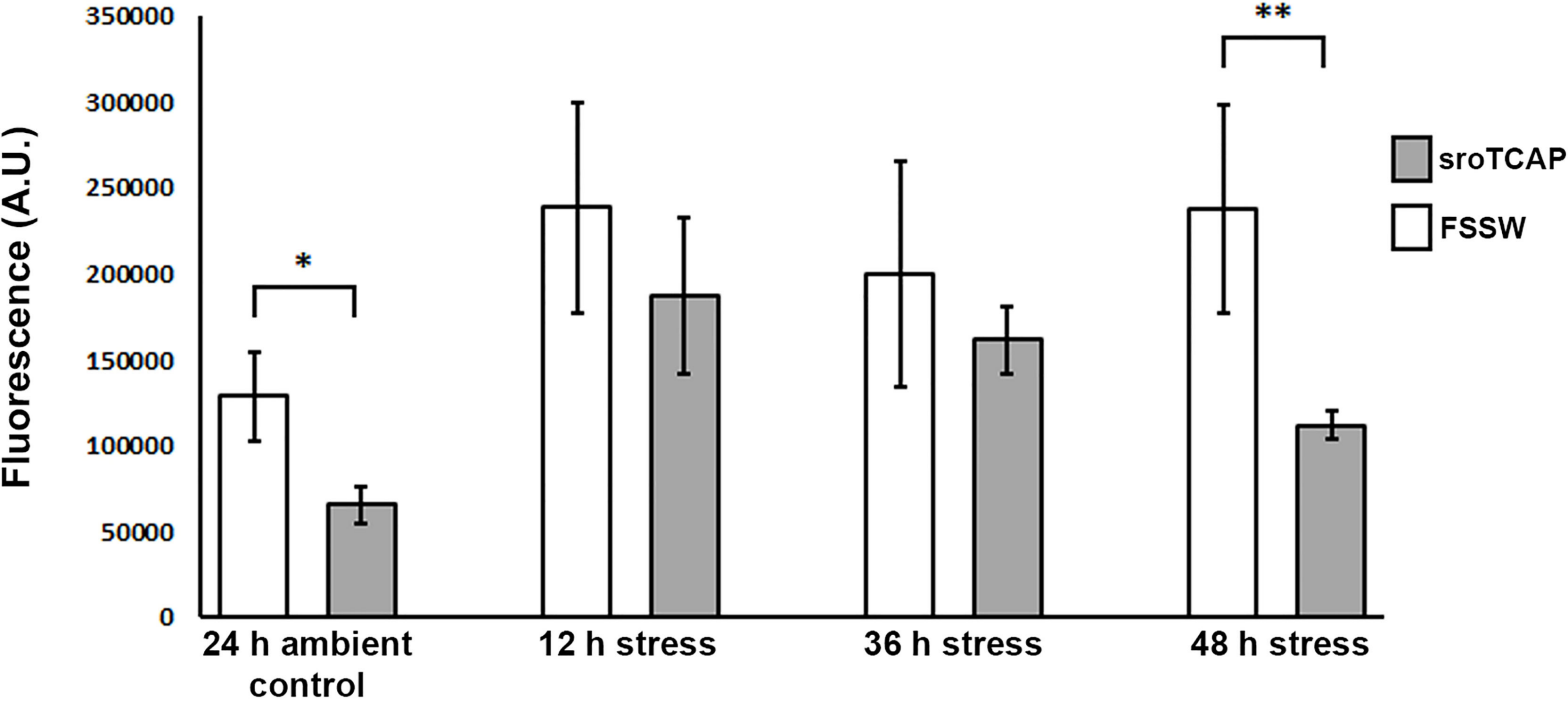
ROS production in SRO hemocytes. Oysters were held in either ambient conditions (35 ppt and 22 ± 1°C) or stress conditions (15 ppt and 30 ± 1°C) for 12 – 48 h, and treated with either sroTCAP (5 pmol) or FSSW (negative control). Y-axis shows the arbitrary units of fluorescence which reflects the ROS activity, higher fluorescence demonstrates higher ROS and vice-versa (*P < 0.05, **P < 0.01).

The effect of sroTCAP on ROS production in hemocytes of stressed oysters was assessed. Peptide (5 pmol sroTCAP) or FSSW was administered to SRO, which were then subjected to ambient conditions (34 ppt and 22°C) for 24 h or stress conditions (15 ppt and 30°C) for 12, 36 and 48 h. Hemocyte ROS production was significantly reduced (P<0.05) in peptide-treated oysters compared to FSSW controls when held in ambient conditions for 24 h (**Figure 6**). During the stress challenge, no significant differences were observed after 12 and 36 h between sroTCAP and FSSW treatments. However, at 48 h, ROS activity had significantly reduced (*P* < 0.01) in peptide-treated SRO compared to the FSSW-treated treatment (**Figure 6**).

### 3.4 Quantitative Transcriptome Analysis of Hemocytes Following sroTCAP Injection, With and Without Stress

**Figure 7:**
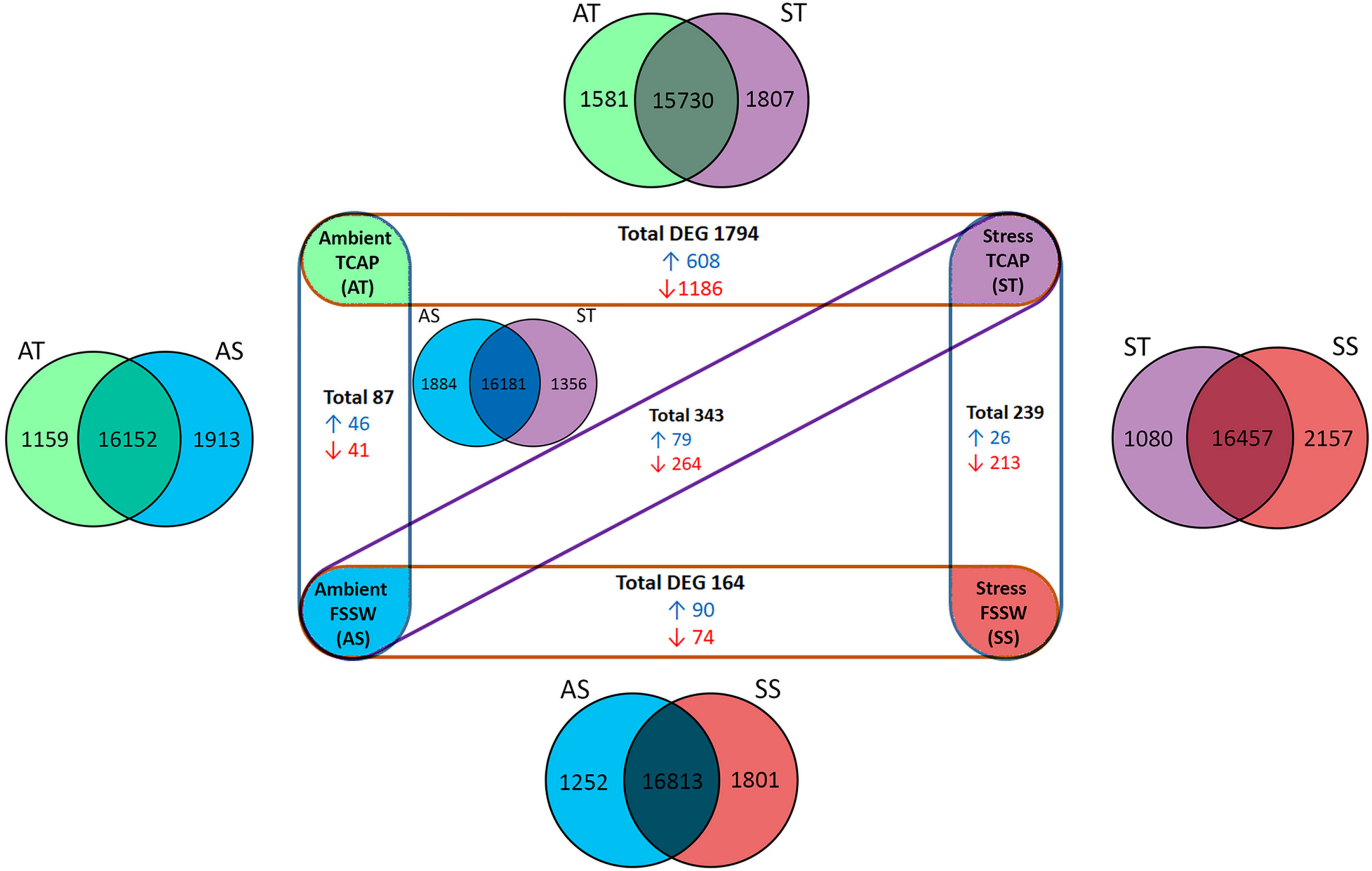
Hemocyte gene expression in response to TCAP/FSSW and ambient/stress treatments (n=4) in different pair-wise comparisons (n=5). Venn diagrams show the total number of expressed genes between different comparisons, including unique and shared expression. Experimental groups show significant DEG of each pair-wise comparison. Blue: upregulated DEG, red: Down-regulated DEG. FDR ≤ 0.05, log fold change ≥ 1 and ≤ -1.

Quantitative hemocyte transcriptomes derived from 4 treatments (in triplicates) were evaluated using five different pair-wise comparisons (**Figure 7**). Initially, the total expressed genes of each treatment were compared, showing the number of common genes expressed between the comparison and the number of unique genes expressed in each treatment (**Figure 7** **- Venn diagrams**). Comparison of the controls (no sroTCAP, SS *vs* AS) indicated a total of 164 DEGs (90 upregulated and 74 downregulated). Comparison of ST *vs* SS (stress with and without sroTCAP administration) indicated that the peptide significantly downregulated 213 genes, while 26 genes were upregulated. The highest number of DEGs was observed for ST *vs* AT, where 1186 genes were downregulated, suggesting that the peptide stimulated widespread transcriptional changes under stress, compared to ambient conditions. DEG data for all comparisons can be found in **Table S4**.

Although not differentially expressed, the mean of *teneurin* expression in hemocytes under stress was higher compared to ambient. Following TCAP administration, *teneurin* slightly decreased compared to corresponding controls **(** **Figure S4** **)**. As the differences in expression were not statistically significant at 3 h post administration, a feedback mechanism cannot be concluded.

**Figure 8:**
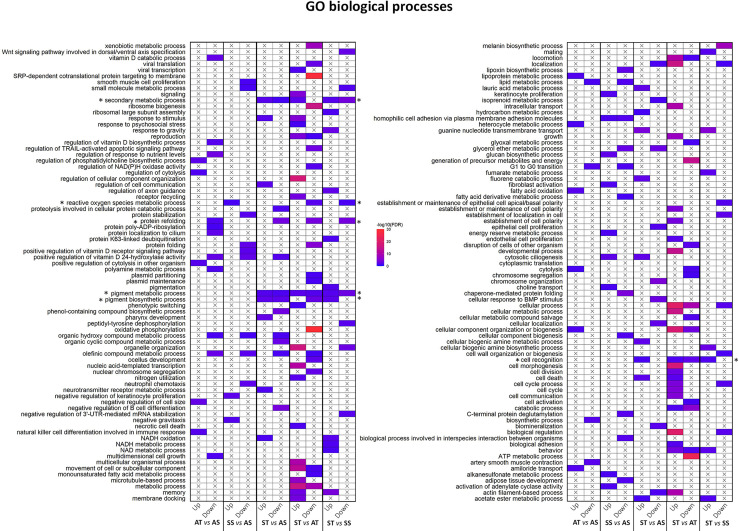
Changes in hemocyte gene expression in response to TCAP/FSSW and ambient/stress treatments. Plots showing the over-represented biological processes in the upregulated and downregulated DEG list in response to TCAP/FSSW and stress/ambient (transcriptome comparison). Each row represents a GO term category, and each column represents the treatment replicates. The most representative and significant biological processes are shown. The colour depicts the significance of the enrichment (-log10 (FDR-corrected *P*-values)). The X mark in the cells means that the GO term is not a representative term in the corresponding DEG list in a comparison. Asterisks represent enriched GO terms of interest.

The DEGs for each comparison was used to obtain a corresponding enriched gene ontology list which was plotted and visualized (**Figure 8**). Overall, enriched GO terms varied depending on the comparisons. However, some GO terms consistently changed when TCAP was administered. For example, protein refolding (GO:0042026), ROS metabolic process (GO:0072593), pigment metabolic process (GO:0042440), pigment biosynthetic pathways (GO:0046148), secondary metabolic processes (GO:0019748), and cell recognition (GO:0008037).

**Figure 9:**
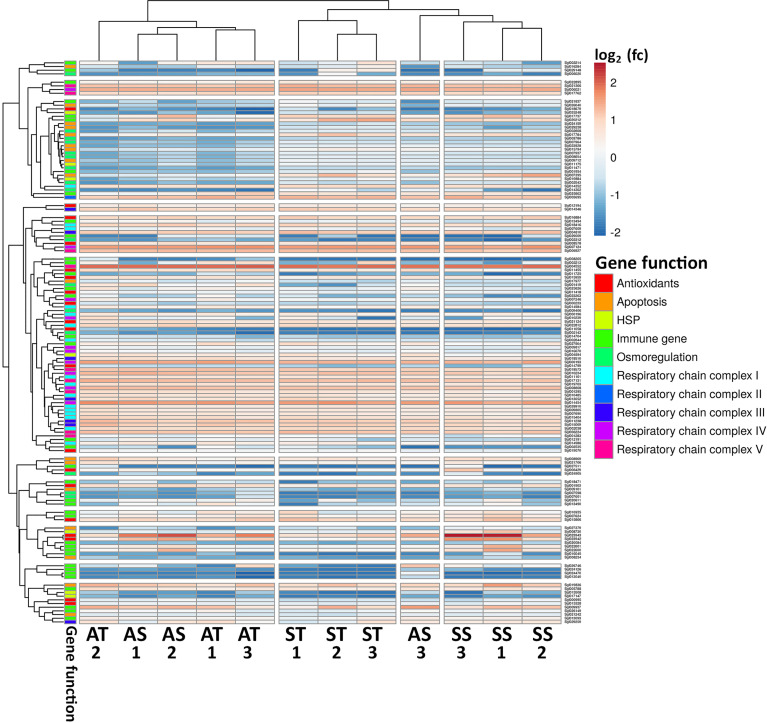
Heatmap showing DGE of genes of interest in several relevant categories, colour coded within gene function. The colour of cells represents the log2 fold change (fc) expression value. Dendrograms of columns and rows show clustering based on average correlation. AS- Ambient condition and FSSW injected, AT- Ambient condition and TCAP injected, SS- Stressed and FSSW injected, ST- Stressed and TCAP injected. Numbers represent the biological replicates of each group (1–3).

A heatmap was prepared showing clustering based on the correlation of gene expression across treatments and including gene ontology functional categories (**Figure 9** **)**. Both peptide and FSSW injected replicates exposed to the ambient conditions (AT and AS) clustered together, suggesting that relatively similar changes occurred at the molecular level. However, AS replicate #3 was an outlier since it clustered closer to SS replicates. All ST and SS replicates clustered separately.

Several osmoregulation and apoptosis inhibitors genes were upregulated in oyster hemocytes under stress and treated with sroTCAP (ST) compared to the other treatments. Most antioxidant genes were downregulated in ST compared to other treatments. HSPs were mainly downregulated in ST. However, a select few seem to have increased their expression compared to the other treatments. Most immune genes were downregulated in ST, with a similar trend in genes involved in the mitochondria chain complexes.

Changes to hemocytes transcripts in SRO treated with TCAP and subjected to stress or ambient conditions showed changes in several groups of genes. This study focused on immune genes, metabolism, and stress-related pathways to better understand the molecular mechanism of action and complement the physiological assays’ data.

### 3.5 Pattern Recognition Receptors

Pattern recognition receptors (PRRs) are major and important parts of the innate immune response. A single *toll-like receptor* gene was differentially expressed and significantly downregulated in both ST *vs* AT and ST *vs* SS groups but was not differentially expressed in the other group comparisons. *Macrophage mannose receptors* were downregulated, with two genes in ST *vs* SS and one in ST *vs* AT. *Peptidoglycan-recognition protein* was downregulated in ST *vs* AT but was upregulated in AT *vs* AS. In ST *vs* AT, five *Interferon-induced protein* transcripts were all downregulated, while in ST *vs* AS, one *Interferon-induced protein* gene was up and downregulated and in SS *vs* AS, a single *Interferon-induced protein* gene was upregulated. Lectin genes such as *lectin BRA-3-like isoform X1, C-type lectin superfamily 17 member A*, *L-rhamnose-binding lectin CSL3 isoform X4* and *fucolectin-like isoform X2* were all downregulated in ST *vs* SS and ST *vs* AT and were differentially expressed (DE) in the other comparisons.

### 3.6 Immune Signalling

Most immune signalling genes were DE in ST comparisons. *Myeloid differentiation primary response protein 88* (MyD88): two isoforms downregulated in ST *vs* AT and one downregulated in SS *vs* AS. *Complement C1q tumor necrosis factor proteins*: downregulated in two ST comparisons where eight genes were downregulated, while a single gene was downregulated in SS *vs* AS. AT *vs* AS was the only comparison having one gene upregulated. *Tumor necrosis factor receptor superfamily member EDAR-like*: was only DE in ST *vs* AT and ST *vs* AS where two genes were upregulated and one downregulated.

In ST vs AT, a single gene of latent-transforming growth factor beta-binding protein 1 isoform X3, interferon regulatory factor 8 isoform X2, receptor-interacting serine/threonine-protein kinase 1, protein pellino-like and big defensin 1 were downregulated and were not DE in other comparisons while two mitogen-activated protein kinase kinase kinase genes (MAP3K) were upregulated.

In ST *vs* AT, three *Nuclear factor erythroid 2-related factor 2-like isoforms* (Nrf2) genes, which are stress-related transcription factors, were upregulated, while in ST *vs* AS, one isoform was up and downregulated.

### 3.7 Antioxidants and Respiration

Antioxidant genes are important cell-protecting mechanisms. Antioxidant DEGs were detected mainly in the ST *vs* AT comparison, where all detected genes were downregulated. These include three *extracellular superoxide dismutase [Cu-Zn]-like* and *superoxide dismutase* genes, while ST *vs* SS had two downregulated *extracellular superoxide dismutase [Cu-Zn]-like* genes. Glutathione-related DEGs were only found in the ST *vs* AT comparison, having two *epididymal secretory glutathione peroxidase-like*, two *microsomal glutathione S-transferase 1*, two *putative glutathione-specific gamma-glutamylcyclotransferase 2*, *glutathione peroxidase-like isoform X1* and *glutathione S-transferase omega-1-like*. ST *vs* AT was the only comparison having downregulated DEG of five *thioredoxin* genes and one *peroxiredoxin* gene. *Alcohol dehydrogenase [acceptor]* was significantly downregulated in AT *vs* AS but was highly upregulated in ST *vs* AT. Overall the antioxidant genes were downregulated when SRO was exposed to stress with TCAP administration, compared to stress with FSSW or ambient conditions with TCAP.

Mitochondrial respiration genes were examined in all comparisons. DEGs were detected only in two comparisons. In AT *vs* AS, *ATP synthase subunit delta* (complex V) was upregulated. Interestingly, ST *vs* AT was the only comparison with downregulated DEGs associated with all five mitochondrial respiration chains (I-V). These include; 16 *NADH dehydrogenase [ubiquinone] subunits* (complex I), *putative succinate dehydrogenase [ubiquinone]* (complex II), seven *cytochrome b-c1 complex subunit* genes (complex III), ten *cytochrome c oxidases* (complex IV) and nine *ATP synthase subunit genes* (complex V) all downregulated.

### 3.8 Apoptosis and Phagocytosis

Several apoptosis inhibitor genes were detected in ST *vs* AT where six different isoforms of *baculoviral IAP repeat-containing protein* were downregulated but had upregulated three *baculoviral IAP repeat-containing protein* and two *apoptosis 1 inhibitor* genes. In SS *vs* AS, a single *apoptosis 1 inhibitor* gene was upregulated. These genes were not DE in other comparisons.

Lysosymal enzymes are proteins involved in phagocytosis (26, 43). ST *vs* SS and ST *vs* AT had downregulated lysosymal enzyme genes. These include; *lysozyme 1-like*, *c-type lysozyme* and *goose-type lysozyme 2*. While in SS *vs* AS, one *lysozyme 1-like* gene was downregulated.

### 3.9 Transcripts Encoding for Heat Shock Proteins

Three types of heat shock proteins were identified as DEG. *Heat shock 70 kDa protein 12A-like*: three genes downregulated in ST *vs* SS, one downregulated in ST *vs* AS and in ST *vs* AT, two were downregulated, and two were found to be upregulated. In both ST *vs* AT and SS *vs* AS, *heat shock protein 68-like* was upregulated while *10 kDa heat shock protein, mitochondrial-like* downregulated.

### 3.10 Osmoregulation

Genes related to osmoregulation were mostly DE and upregulated in comparisons with ST. Four genes of *ATP-binding cassette sub-family A* and *C members* were upregulated, while in SS *vs* AS a single gene was upregulated. Two *solute carrier family 12-member* genes were upregulated in ST *vs* AT only. *Monocarboxylate transporters* were only DE in three ST comparisons where four were upregulated and three were downregulated.

## 4 Discussion

As an intertidal species, the SRO is constantly exposed to various environmental stressors. Therefore, understanding TCAP’s ability to improve the stress response is desirable. In this study, we aimed to understand the impact of sroTCAP on the hemocyte stress response. The finding provides new knowledge into TCAP function in an invertebrate species.

### 4.1 SRO Teneurin Shows Similarity to Vertebrate Teneurins

In this study, the gene encoding for teneurin was identified and characterized in the SRO, utilizing considerable genomic and transcriptomic resources (26, 28, 31, 36). A single *teneurin* gene was identified in the SRO genome, consistent with findings in the *C. elegans* and *C. intestinalis* (19, 44), while most vertebrate genomes possess four orthologues (6, 44). Transcriptomic analysis demonstrated that SRO has four *teneurin* isoforms that encode precursor proteins ranging from 2776 to 2807 amino acids. This is also consistent with what was determined for other *teneurin* genes, where splice variants or modified post-translational proteolytic cleavage leading to smaller precursors are commonly found (10, 45). All SRO teneurin precursors had a conserved dibasic ‘RR’ predicted cleavage site that could liberate an identical TCAP from the C-terminus.

SRO *teneurin* was found to be expressed at varying levels in all oyster tissues analyzed, which aligns with *teneurin* expression data for vertebrate species, showing expression in neural and non-neural tissues such as muscle, gonads (46), eyes, limb buds, heart, skin (45) and dental pulp (47), suggesting a role in cell migration and pattern formation (48). Across commonly studied bivalves, TCAP has a conserved P-E-L-S-D motif, which is similar to the conserved P/L-E-L-A/S/T-D motif found in vertebrate species (11). This conservation was supported at the structural level, with a predicted 3D structure model of sroTCAP exhibiting high identity to the human TCAP-2 structure, which implies a similarity of function between both species.

### 4.2 TCAP Is Distributed Across Tissues With Variable Cleavage Patterns

In order to observe the presence of sroTCAP in various tissues, an anti-sroTCAP antibody was produced. Anti-TCAP antibodies were used in western blots, where multiple ir-TCAP bands were detected in mouse brain extract and E14 cell lines, including a large ~70 kDa protein (10) and in multiple tissues of *C. intestinalis* (19). We also observed multiple ir-TCAP bands in SRO protein extracts, with the most prominent band found at ~22 kDa in most tissues and a ~37 kDa band detected in muscle, hemocytes and hemolymph. The ~37 kDa protein is most likely due to alternate teneurin cleavage. TCAP (or teneurin processed derivatives) in SRO cell-free hemolymph provides the first evidence that it circulates in the serum of animals. We predict that if a putative dibasic cleavage site was used in the SRO teneurin, the released TCAP would be ~ 7.09 kDa (60 amino acids). However, unlike in *C. intestinalis*, no ir-TCAP was detected at that size in SRO, suggesting the cleavage site was either non-functional or the liberated TCAP formed multimers. The latter is consistent with our observation that synthesized sroTCAP (both 42 and 60 amino acid lengths) appears mainly as dimers and trimers in a western blot. The formation of multimers were also shown to occur in synthetic mouse TCAP-1 (49). Therefore, the ~22 kDa ir-TCAP band observed in SRO tissues could be a TCAP trimer. A similar observation was made in mouse E14 cell lysate and mouse brain peptide extract, where the expected 4.7 kDa mouse TCAP peptide was not detected (10). A 15 kDa band (approximately three times larger) was detected, suggesting that TCAP may exist as a trimer endogenously (10). An alternative explanation could be proteolytic cleavage upstream of our predicted TCAP sequence (5 putative alternate proteinase cleavage sites were identified) (**Supplementary Table S3**).

### 4.3 TCAP Localizes in the Cytosol and Strongly in Granules of Hemocytes and Is Associated With Stress and Apoptosis Modulation

SRO TCAP was observed in the cytosol and granule-like bodies of hemocytes. No membrane-bound ir-TCAP was detected, suggesting that TCAP is cleaved post-translationally before or after teneurin is embedded in the outer membrane. A TCAP processing mechanism was previously proposed by Chand, Casatti (10) in a mammalian model, where the full-length teneurin was translated as part of the secretory pathway, and the TCAP was liberated in the secretory vesicles or on the plasma membrane. Overall, based on our sequence, western blot and immunohistochemistry analyses, our results suggest that sroTCAP is generated as part of a teneurin precursor, then processed to function independently, as previously described in vertebrates species (10).

To further understand the mechanism of action of sroTCAP, we investigated potential binding proteins using a pull-down assay. Under the highest stringency conditions, we identified a binding affinity with EcSOD and GAPDH. Following TCAP administration to mouse neural cells under stress, SOD levels were shown to increase (18). However, the functional significance of this binding interaction is still unclear. In *C. gigas*, EcSOD can mediate the attachment and invasion of *Vibrio splendidus* to hemocytes *via* an outer membrane protein (OmpU), where EcSOD acts as an opsonin, promoting phagocytosis (50). Thus, sroTCAP binding to EcSOD may be involved with blocking the binding to OmpU, thereby limiting infection.

**Figure 10:**
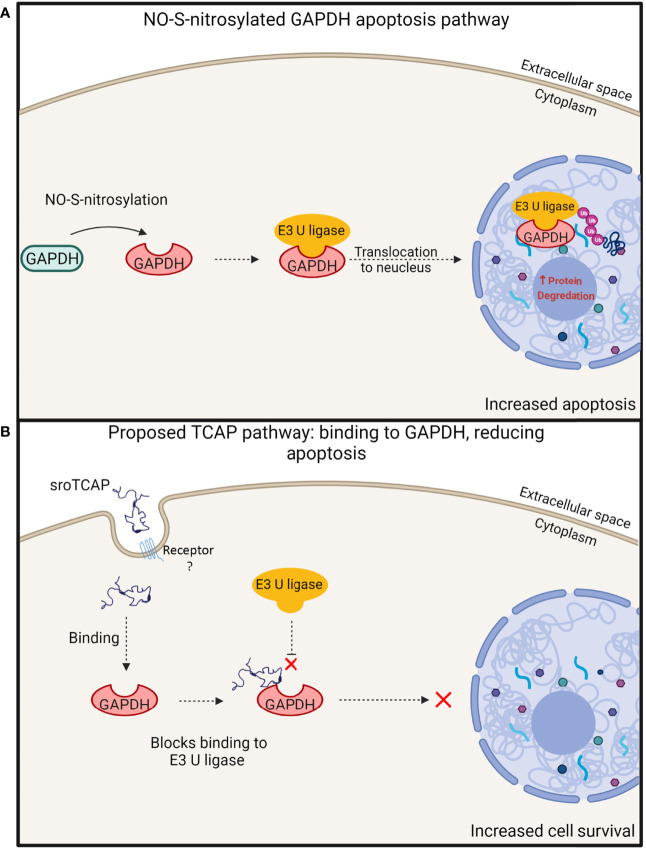
Proposed model of TCAP and GAPDH interaction, leading to increased cell survival during stress. **(A)** Under oxidative conditions, GAPDH undergoes NO-S-nitrosylation, where it can then bind to E3 ubiquitin ligase. This protein complex transits to the nucleus, degrading nuclear proteins, resulting in apoptosis [modified from Hara et al. (51)]. **(B)** A proposed TCAP interaction with GAPDH leading to the inhibition of GAPDH binding with E3 ubiquitin ligase, thus preventing its downstream apoptotic actions. The inhibition of this pathway supports increased cell survival under stress. Created with BioRender.com.

A potential sroTCAP interaction with GAPDH is consistent with the results by Trubiani, Al Chawaf (18), where TCAP administration to mouse hypothalamic cells *in-vitro* during cellular stress reduced apoptosis and increased cell viability by 5-fold, due to binding with GAPDH. During cellular oxidative stress, GAPDH can undergo nitric oxide (NO)-S-nitrosylation that changes its conformation and enables it to bind to E3 ubiquitin ligase (vertebrate homologue –Siah1) (51, 52). E3 ubiquitin ligase contains a nuclear localization sequence that enables the complex with GAPDH to translocate into the nucleus (51, 52). GAPDH acts to stabilize E3 ubiquitin ligase while it degrades nuclear proteins, leading to apoptosis (51, 52) (**Figure 10A**). We hypothesize that the binding of TCAP to GAPDH may inhibit its interaction with E3 ubiquitin ligase, preventing nuclear translocation and apoptosis (**Figure 10B**).

The ability of TCAP to bind to multiple proteins suggests it may be a moonlighting protein, where a single protein could perform multiple functions depending on its cellular, intracellular or extracellular location and ligand/substrate concentration [reviewed by Jeffery (53, 54)]. Interaction of moonlighting proteins with different partners can affect their structure and therefore binding affinities and actions, especially for intrinsically unstructured proteins [reviewed by Tompa, Szász and Buday (55)]. Li et al. suggested that after TCAP cleavage from teneurin, it undergoes major structural changes (56), supporting our hypothesis that liberated TCAP has moonlighting properties.

### 4.4 Low Dose sroTCAP Modulates Phagocytosis and ROS Under Stress

In oysters, hemocyte phagocytosis activity is a marker commonly used to evaluate immune response, which has been shown to be reduced under prolonged stress and, therefore, increases the risk of infection and mortality (22, 22, 26, 57, 58). In this study, high temperature (30°C) and low salinity (15 ppt) stressors were used to determine the effect of sroTCAP on hemocyte phagocytosis. Under non-stressed, ambient conditions, hemocytes exhibited considerably reduced phagocytic activity following sroTCAP administration compared to controls. Under stress conditions, the phagocytosis activity of the control groups increased significantly at 3 and 12 h post-stress. Under the same stress conditions, sroTCAP administration was able to reduce the level of hemocytes phagocytosis activity similar to that found in ambient controls.

ROS play a major role in phagocytosis as part of oyster’s lytic defense mechanisms and are produced as part of normal metabolism, in addition to internal and external stimuli (59). Hemocytes produce ROS through several hydrolytic enzymes to inactivate and destroy ingested foreign particles (60, 61). If ROS production is prolonged, it can cause hemocyte cytotoxicity and apoptosis (62). Therefore, the ability of sroTCAP to regulate hemocyte ROS abundance at ambient and stress conditions was investigated. We found that 5 pmol of sroTCAP significantly reduced ROS after 48 h of stress conditions compared to the FSSW control. This result agrees with Trubiani et al. (18), wherein mouse hypothalamic cell line (N38) exposed to stress for 48 h post TCAP delivery had increased the expression of SOD and catalase, thereby reducing ROS and protecting the cells from oxidative damage. Our data indicates that TCAP caused a significant reduction of ROS, not only under stress but also under ambient conditions in hemocytes at 24 h post-treatment.

Over time, it was observed that sroTCAP treated groups under stress had a decline in ROS production, which indicates an upregulation of free radical scavenging mechanisms, a reduction in enzymatic ROS production pathways, or a combination of both. The reduced phagocytosis and the subsequent reduction of ROS in TCAP treatment under both stress and ambient conditions are interrelated, as both are a factor of hemocyte activity (62). This suggests that TCAP action on hemocyte activity occurs upstream in these immune/stress-related pathways. A review by Kazutaka and Keisuke (62) demonstrated that apoptosis could be triggered in oyster hemocytes, not only by ROS production but also by phagocytosis, which implies that TCAP can protect hemocytes from apoptosis under stress. The protection of hemocytes is crucial, especially under stress, due to their involvement in several important biological functions outside of innate immunity, such as the formation and repair of both tissues and shell, nutrient transport and maintaining homeostasis (63–66), which are essential and advantageous for combating stress.

### 4.5 sroTCAP Modulates Key Protective Genes Under Stress

To better understand sroTCAP effects on hemocytes, a quantitative transcriptome analysis was conducted following sroTCAP injection, with and without stress. Overall, only a small fraction of genes was expressed uniquely in each treatment group, with most genes being expressed concurrently in each pair-wise comparison. However, we found that administration of sroTCAP consistently affected biological functions related to protein refolding, pigment metabolic process and biosynthetic pathway, secondary metabolic processes and cell recognition. This points to sroTCAP having additional associated functions to immune, metabolism and stress response functions, which were the focus of this study and described in the following sections.

Reactive oxygen species metabolic process, which involve biological pathways of ROS production, was downregulated in stressed with TCAP comparisons but upregulated in the stressed with FSSW control comparison. As mentioned, a significant reduction in measurable ROS was only apparent at 48 h post-TCAP treatment. However, a decrease in the ROS production pathway in the hemocyte transcriptome data was detectable 3 h post-TCAP treatment. This may explain the observed decrease in ROS over time in the TCAP treated group under stress and the ROS increase in the FSSW treated group.

The significantly differentially expressed genes following TCAP/stress treatments show that several pathways and mechanisms were modulated by TCAP administration, which are potentiated when SROs were subjected to stress. A similar observation was seen *in-vitro* in mouse hypothalamic cells, where it was shown that some protective proteins changed significantly only in TCAP treatment under stress (18).

Immune-related pathways, including PRRs, immune signaling, and phagocytosis genes, were mainly downregulated in TCAP injected stressed SROs, consistent with our results showing that phagocytosis activity did not increase when stressed SROs were treated with TCAP. Complex I and III are the main ROS generators in the mitochondria (67), and inhibiting complex I by RNAi was shown to reduce ROS production and apoptosis in human T-cells (68). TCAP administration followed by stress downregulated the expression of all five mitochondrial complexes compared to TCAP at ambient. This observation supports the claim that the decrease in ROS over time when SROs were treated with TCAP in both stress and ambient conditions is most likely due to a reduction in the mitochondrial ROS production and not the upregulation of antioxidant genes. Under stress, TCAP administration reduces ROS production in hemocytes, most likely through a significant decrease in mitochondrial activity compared to TCAP administration under ambient conditions. Furthermore, several apoptosis inhibitor genes were DE in ST *vs* AT, suggesting that TCAP may regulate stress-induced apoptotic pathways.

Two heat shock proteins (*HSP 70 12A-like* and *HSP 10kDa*) were mostly downregulated in stressed, TCAP treated groups compared to the other treatments. HSPs are important protective proteins, present at normal physiological conditions but upregulated during stress (69). HSPs protect proteins from damage and irreversible denaturation, however, these proteins are also known to modulate the immune system by various pathways, including the interactions of TLR and PRRs (70). HSPs and antioxidants contribute to cellular protection and defense against a plethora of molecular and physiological stresses (2, 69). Therefore, it is interesting that these protective genes are predominantly downregulated under stress with TCAP administration while upregulated or unchanged at ambient conditions post TCAP administration. This indicates that TCAP may lead to a dampening effect of the stress response during abiotic stresses and delay the stress response in SRO, thereby conserving energy in the event that the stress is transient.

In summary, our findings in SRO suggest a molecular role for TCAP in energy metabolism and energy conservation under stress by affecting multiple pathways in hemocytes. It can be reasoned that TCAP may target a master regulator, which triggers the observed effects attributed to TCAP. A possible target is Nrf2, where three isoforms were significantly upregulated in ST *vs* AT. Nrf2 is a transcription factor that regulates many stress-related genes associated with cell homeostasis, cytoprotection, anti-inflammatory response, and innate immunity (71–73). Nrf2 also regulates the expression of several cellular antioxidant genes, including *SOD*, *glutathione peroxidase*, *thioredoxin*, and *glutathione S-transferase* (71, 73), all of which were DE in most ST comparisons.

Oysters in the intertidal zone are exposed to continuously changing environments (74), triggering a stress response due to a brief stress exposure may not be advantageous long term for energy conservation. Thus, although counterintuitive, preventing an increase in the stress response during acute periods of stress could benefit energy conservation and longevity. Further studies are needed to investigate whether the reduced molecular stress response provides physiological protection to SRO and whether the response induced by TCAP is protective against pathogens.

In marine animals, the regulation of energy expenditure is critical for survival under stress conditions (75, 76). TCAP seemingly has a role in metabolic adaptation in oysters, as the reduction of immune activity, hemocyte ROS, and mitochondrial activity demonstrate its ability to potentially allow for the deviation of energy to areas where it is needed most for survival under stress.

## 5 Conclusions

This study characterized the SRO TCAP, derived from the *teneurin* gene, through analysis of peptide distribution, function and relevant molecular factors. Most interestingly, we have demonstrated that TCAP can modulate stress-related functions, including immune activity in hemocytes *via* regulation of phagocytosis and reduction in ROS levels at ambient conditions and during stress. In SRO exposed to stress, TCAP significantly reduced the expression of genes associated with several protective, metabolic and immune pathways. Notably, a reduction in expression of immune-related genes, HSPs, antioxidant genes, and genes involved in five mitochondrial complexes while upregulating genes involved in osmoregulation and apoptosis inhibition. We speculate that some effects attributed to TCAP may be due to the activation of Nrf2 under physiological stress. TCAP effects were potentiated when SRO was subjected to stress as opposed to ambient conditions. These findings suggest an important role of TCAP in stress regulation and metabolism in an invertebrate and may have important implications for higher-order metazoans.

## Data Availability Statement

The datasets presented in this study can be found in online repositories. The names of the repository/repositories and accession number(s) can be found below: https://www.ncbi.nlm.nih.gov/, PRJNA804582.

## Ethics Statement

Ethics review and approval was not required as per local legislation and institutional requirements.

## Author Contributions 

AE, LP, and DL conceptualized the manuscript. TA, SS, PS, SC, DL, and AE contributed to the design of the manuscript. TA, SS, and PS conducted the experiments. TW contributed to the LC-MS/MS analysis. TA, SS, SC, and AE wrote the manuscript. MD and WO’C provided the experimental animals. All authors contributed to the article and approved the submitted version.

## Funding

Cooperative Research Centre Project (CRC-P 2016-553805; Future Oysters) to MD, WO’C, AE; ARC Discovery Indigenous (IN190100051) to LP, AE; and USC PhD scholarship for TA.

## Conflict of Interest

The authors declare that the research was conducted in the absence of any commercial or financial relationships that could be construed as a potential conflict of interest.

## Publisher’s Note

All claims expressed in this article are solely those of the authors and do not necessarily represent those of their affiliated organizations, or those of the publisher, the editors and the reviewers. Any product that may be evaluated in this article, or claim that may be made by its manufacturer, is not guaranteed or endorsed by the publisher.

## Abbreviations

TCAP, Teneurin C-terminal Associated Peptide; SRO, Sydney Rock Oyster; sroTCAP, TCAP protein sequence of SRO; ir, Immunoreactive; aa, Amino acid; FSSW, Filter Sterilized Sea Water; IM, Intramuscular; ROS, Reactive Oxygen Species; DEG, Differentially Expressed Genes; DE, Differentially expressed; PRR, Pattern Recognition Receptor; TLR, Toll-Like Receptor; HSP, Heat Shock Protein; Nrf2, Nuclear factor erythroid 2-related factor 2.
