# Supplementary material for: Teneurin and TCAP Phylogeny and Physiology: Molecular Analysis, Immune Activity, and Transcriptomic Analysis of the Stress Response in the Sydney Rock Oyster (Saccostrea glomerata) Hemocytes
Source: Front Endocrinol (Lausanne). 2022 Jun 17;13:891714. doi: 10.3389/fendo.2022.891714 (PMC9248207; doi:10.3389/fendo.2022.891714)
Supplement: Supplementary file 1 [file DataSheet_1.docx]

**Supplementary Table 1.** Predicted SRO teneurin isoforms detected in transcriptome data.

| **SRO Teneurin isoform** | **Protein sequence** |
| --- | --- |
| SroTEN-X1_ID\|364862 | MQVQTGPAMDSNYNYRPKGQGQSQRKRCLKDSRMNSSQGSCSSDEEFHSDDNLRPYEEVKV  AHQDKKLNGLGLTPEEHVELTAMDQKVHMMCSKNNNKTPRFYTYREGSEGEESEQNLSV  STKHASLSVNHQGSQCSISSNEMDNMHSMRNEVVSRSDSDPDNQNHHINSPLGQNKFHHA  ATAPGGLPPCFNPPPPPPPIEDIPHQTTVPQRLNRCMGPSYRGTYSDADMCQCRGHFVDS  NTALLHNHHSIRGHYSDNENPYHRGHSCSDSEHYHQVRPCVYSGEESDFEPHYLEQTASG  NVFIPDGQPRFPRTVPRSGSVPMSRSGELVSPQPIRPQCSNTVPSHFLNGEHHNSERQAF  LPHGQCGYPANMTSPPRSHDCGYTKFGNTPHYMKKKLKRWSWKWAALILVIICVGLLAAT  TYFAAHLTFEKNEKKEEVPGALNHTVLKFTQTPFHLDGSRPTYTTIPPLTLWQTYFNQKD  SKFVKFDFTIPSSARLAIYGRRNFPPTIAQFDFYQVYDGSTIKDPSAQTMRDRYKRSLPD  KQTALVQMMEKGKWYITIYNDRDQPQEIGVSRTTIDSMGSSCERDCFGHGLCDNQGRCQC  FNGYRGPYCSELECPRLCNGQGEYRQGVCVCHEGWKGPECDTPANKCENPTCNNRGRCVD  GQCQCDKGFTGPHCGIVTCIDPSCSGNGLCHLGKCVCYKGFKGDNCQLPDKLNLTHLCAR  DCSGHGQFDWDTGRCVCHRFFTGRDCEQEMCRLRCINGRCHNQRCVCDKGWGGVLCDTRN  CDPRCDGVKGQCDKGTCICRTGWNGKHCTIDGCPNGCSNHGNCRRYGNSGYKCDCHAGWK  GSGCNIAMEMMCSNNVDDDNDGLSDCLDPDCCSSAACTRSPFCQTVQDPAEILLQKPKPS  STASFFKKMKFLIDNNSIQKETSKNAFNESQVSVIRGHVETRDGTPLVGVRVNVRIQPLY  GHTLTRNDGRFDILVNGGGSVTLEFTRQPFQSHTISVSVPWNQIITMETVIMDLQSGDFT  EPDPSLCGVGHDHHSMKPIVLSTWQHTQLGACPEKSTLIPESQVLQESVEVPGTNVHLVY  HSSETEGYKSVILIQMTPDTIPTNLALVHLKVYVQGVETVKVFEADPGLKYTFSWDRINA  YRQKVYGIVPVKVHVGYEYKGCTYVFWEVRSTTMTGFDLTSSEIGGWNLDIHHTYNFQEG  ILHKGDGSNIYLKEKPQELVCILGNGIQRKLDCAMCNGDASNNQVRAPVALASGSDGSLY  IWDDNFIRKLSPGRTEIVSILKTDSVFHKTYMTVSPVNGKLYISDYMHHRVIQIATMGPV  QNLEQNFKVIAGNGEECSTGLLDECGDGGLAIQARLLGPKGIAINKEGVIYIADNLNIRQ  ISSTGIITTLIGSHNQLRTQEPMSCDHSRPANQVQLHWPTALAIDPLDDSLHILDKNVIL  KLTKDNYIVTIAGRPSNCPIRSIGSLLSGILSDEEEASGIAAEVRLVDAQSITFGPHGEI  HVVESDQHRINRVRVITSDGRIHHFAGSKSKCDCKSKTCLCYDAKETLAAQALFNSLTSI  TVTPDGIVHIADNGNLRVFSIMSKLPQPDTNNKYKVYSPDTKEMYIFNDHGQHQQTVDIM  TGQYMYNFTYNVNSFFSKLVSVTDDIKNMIELTRDSNLQVTQVISPGNQRSKVEMNNLHR  LQRFTSPNNNSLSFTYKGTTGLLESKYLSNGQSYFYNYNDKGRLMETRQPTGEITSLVTD  INTTGSIVRVNTDSSDVISMATYGSVQSVMHVKISQGAAETQVTYLPDGGVVVMYPTNMS  ITIESGGHPVLSNQHRMHFKRKIIGPNQLVHKLEWRFYARRRYSPSCGRKTLQRLGSKMR  VGESRPHVSMSDSPDMSSRGEAKALRINGVNLLSVEYDRVNHTESILNKDSQNILWIMYD  DSGLPVQFLPCSEHHAMNITYNQRGQITHWQYGEMWEDLNYNRDGLLLERSRSGTVQYRF  NYRYGKSSPTDIVMPSGKQYYLEYNSLGELEKIRTPDLGYHHFNHITSIGKQRYLYHIPG  LSYPYSEEYDGNGKLLMLVYPSEQRRVAYRYNSYSQPTMVLFDETQVELEYNEQILKLSQ  STISSGPYSCVETYAYSGSLVSSYDVTFPKDDKLITGSFSYTYDDNFRITKIDAAFGVHI  NGTSTSYAYDTNTGKLKTLGPLNLTFRTMYDSETISDKYVTVTRSYDKYGRVENTKYRFD  RDSVLTLKVGYDVYNRIHRWQRNVNGEEIKYMYMYDKDSNIIEVFINGQSAWRFSYSNNG  NINRVTEDGIPKDLEYDTGDRIIKSGNKQYKFDEDGFMAKRHDQDLKFNSNGQLMYVAKT  GKYRYFYFYDSFGKLVLMESNGGETMQYFYSDVSNPNRITHTYNRTSLEVTEYIYEPSGS  LIAMTRGGLVYYIACDPMGSPIAVMNKQGHIIKSIVYDPLGRVENDTNPSFEFSFGFQGG  LYNPVTELVIFSSRVYDTDNGRWLSPSYKNIIHNIQKILEAPALLNNYRFQYLVNTHTKT  SYPILSVTEWMSMLGYDIRSLAPDVSYTGEIRPKKKDTDLSLLPTSSAFECTFLQDMDSL  LTMSIVPKSKLSPLQTRTDVRFAALGSIFGDGVTLSYRNGHVEVGVMEKTPDWSKQLALV  LVNGSEILDLQYIINGKDVHYFVKPESSKAGEDLKTLGIYNDEIRYENGLNVTVKRPTHR  RTETDIKLHGKHSIINIRYGTSLELERQRVLSHATTRAVNHAWRREKWILQNSLTSQYQW  TSYEVNEILTHGSARGYEGRYRQSQTPTEYPELSDDCNSIKLQKTNR |
| >SroTEN-X2_ID\|364847 | MQVQTGPAMDSNYNYRPKGQGQSQRKRCLKDSRMNSSQGSCSSDEEFHSDDNLRPYEEVK  VAHQDKKLNGLGLTPEEHVELTAMDQKVHMMCSKNNNKTPRFYTYREGSEGEESEQNLSV  STKHASLSVNHQGSQCSISSNEMDNMHSMRNEVVSRSDSDPDNQNHHINSPLGQNKFHHA  ATAPGGLPPCFNPPPPPPPIEDIPHQTTVPQRLNRCMGPSYRGTYSDADMCQCRGHFVDS  NTALLHNHHSIRGHYSDNENPYHRGHSCSDSEHYHQVRPCVYSGEESDFEPHYLEQTASG  NVFIPDGQPRFPRTVPRSGSVPMSRSGELVSPQPIRPQCSNTVPSHFLNGEHHNSERQAF  LPHGQCGYPANMTSPPRSHDCGYTKFGNTPHYMKKKLKRWSWKWAALILVIICVGLLAAT  TYFAAHLTFEKNEKKEEVPGALNHTVLKFTQTPFHLDGSRPTYTTIPPLTLWQTYFNQKD  SKFVKFDFTIPSSARLAIYGRRNFPPTIAQFDFYQVYDGSTIKDPSAQTMRDRYKRSLPD  KQTALVQMMEKGKWYITIYNDRDQPQEIGVSRTTIDSMGSSCERDCFGHGLCDNQGRCQC  FNGYRGPYCSELECPRLCNGQGEYRQGVCVCHEGWKGPECDTPANKCENPTCNNRGRCVD  GQCQCDKGFTGPHCGIVTCIDPSCSGNGLCHLGKCVCYKGFKGDNCQLPDKLNLTHLCAR  DCSGHGQFDWDTGRCVCHRFFTGRDCEQEMCRLRCINGRCHNQRCVCDKGWGGVLCDTRN  CDPRCDGVKGQCDKGTCICRTGWNGKHCTIDGCPNGCSNHGNCRRYGNSGYKCDCHAGWK  GSGCNIAMEMMCSNNVDDDNDGLSDCLDPDCCSSAACTRSPFCQTVQDPAEILLQKPKPS  STASFFKKMKFLIDNNSIQKETSKNAFNESQVSVIRGHVETRDGTPLVGVRVNVRIQPLY  GHTLTRNDGRFDILVNGGGSVTLEFTRQPFQSHTISVSVPWNQIITMETVIMDLQSGDFT  EPDPSLCGVGHDHHSMKPIVLSTWQHTQLGACPEKSTLIPESQVLQESVEVPGTNVHLVY  HSSETEGYKSVILIQMTPDTIPTNLALVHLKVYVQGVETVKVFEADPGLKYTFSWDRINA  YRQKVYGIVPVKVHVGYEYKGCTYVFWEVRSTTMTGFDLTSSEIGGWNLDIHHTYNFQEG  ILHKGDGSNIYLKEKPQELVCILGNGIQRKLDCAMCNGDASNNQVRAPVALASGSDGSLY  IWDDNFIRKLSPGRTEIVSILKTDSVFHKTYMTVSPVNGKLYISDYMHHRVIQIATMGPV  QNLEQNFKVIAGNGEECSTGLLDECGDGGLAIQARLLGPKGIAINKEGVIYIADNLNIRQ  ISSTGIITTLIGSHNQLRTQEPMSCDHSRPANQVQLHWPTALAIDPLDDSLHILDKNVIL  KLTKDNYIVTIAGRPSNCPIRSIGSLLSGILSDEEEASGIAAEVRLVDAQSITFGPHGEI  HVVESDQHRINRVRVITSDGRIHHFAGSKSKCDCKSKTCLCYDAKETLAAQALFNSLTSI  TVTPDGIVHIADNGNLRVFSIMSKLPQPDTNNKYKVYSPDTKEMYIFNDHGQHQQTVDIM  TGQYMYNFTYNVNSFFSKLVSVTDDIKNMIELTRDSNLQVTQVISPGNQRSKVEMNNLHR  LQRFTSPNNNSLSFTYKGTTGLLESKYLSNGQSYFYNYNDKGRLMETRQPTGEITSLVTD  INTTGSIVRVNTDSSDVISMATYGSVQSVMHGAAETQVTYLPDGGVVVMYPTNMSITIES  GGHPVLSNQHRMHFKRKIIGPNQLVHKLEWRFYARRRYSPSCGRKTLQRLGSKMRVGESR  PHVSMSDSPDMSSRGEAKALRINGVNLLSVEYDRVNHTESILNKDSQNILWIMYDDSGLP  VQFLPCSEHHAMNITYNQRGQITHWQYGEMWEDLNYNRDGLLLERSRSGTVQYRFNYRYG  KSSPTDIVMPSGKQYYLEYNSLGELEKIRTPDLGYHHFNHITSIGKQRYLYHIPGLSYPY  SEEYDGNGKLLMLVYPSEQRRVAYRYNSYSQPTMVLFDETQVELEYNEQILKLSQSTISS  GPYSCVETYAYSGSLVSSYDVTFPKDDKLITGSFSYTYDDNFRITKIDAAFGVHINGTST  SYAYDTNTGKLKTLGPLNLTFRTMYDSETISDKYVTVTRSYDKYGRVENTKYRFDRDSVL  TLKVGYDVYNRIHRWQRNVNGEEIKYMYMYDKDSNIIEVFINGQSAWRFSYSNNGNINRV  TEDGIPKDLEYDTGDRIIKSGNKQYKFDEDGFMAKRHDQDLKFNSNGQLMYVAKTGKYRY  FYFYDSFGKLVLMESNGGETMQYFYSDVSNPNRITHTYNRTSLEVTEYIYEPSGSLIAMT  RGGLVYYIACDPMGSPIAVMNKQGHIIKSIVYDPLGRVENDTNPSFEFSFGFQGGLYNPV  TELVIFSSRVYDTDNGRWLSPSYKNIIHNIQKILEAPALLNNYRFQYLVNTHTKTSYPIL  SVTEWMSMLGYDIRSLAPDVSYTGEIRPKKKDTDLSLLPTSSAFECTFLQDMDSLLTMSI  VPKSKLSPLQTRTDVRFAALGSIFGDGVTLSYRNGHVEVGVMEKTPDWSKQLALVLVNGS  EILDLQYIINGKDVHYFVKPESSKAGEDLKTLGIYNDEIRYENGLNVTVKRPTHRRTETD  IKLHGKHSIINIRYGTSLELERQRVLSHATTRAVNHAWRREKWILQNSLTSQYQWTSYEV  NEILTHGSARGYEGRYRQSQTPTEYPELSDDCNSIKLQKTNR |
| SroTEN-X3_ID\|364855 | MQVQTGPAMDSNYNYRPKGQGQSQRKRCLKDSRMNSSQGSCSSDEEFHSDDNLRPYEEVK  VAHQDKKLNGLGLTPEEHVELTAMDQKVHMMCSKNNNKTPRFYTYREGSEGEESEQNLSV  STKHASLSVNHQGSQCSISSNEMDNMHSMRNEVVSRSDSDPDNQNHHINSPLGQNKFHHA  ATAPGGLPPCFNPPPPPPPIEDIPHQTTVPQRLNRCMGPSYRGTYSDADMCQCRGHFVDS  NTALLHNHHSIRGHYSDNENPYHRGHSCSDSEHYHQVRPCVYSGEESDFEPHYLEQTASG  NVFIPDGQPRFPRTVPRSGSVPMSRSGELVSPQPIRPQCSNTVPSHFLNGEHHNSERQAF  LPHGQCGYPANMTSPPRSHDCGYTKFGNTPHYMKKKLKRWSWKWAALILVIICVGLLAAT  TYFAAHLTFEKNEKKEEVPGALNHTVLKFTQTPFHLDGSRPTYTTIPPLTLWQTYFNQKD  SKFVKFDFTIPSSARLAIYGRRNFPPTIAQFDFYQVYDGSTIKDPSAQTMRDRYKRSLPD  KQTALVQMMEKGKWYITIYNDRDQPQEIGVSRTTIDSMGSSCERDCFGHGLCDNQGRCQC  FNGYRGPYCSELECPRLCNGQGEYRQGVCVCHEGWKGPECDTPANKCENPTCNNRGRCVD  GQCQCDKGFTGPHCGIVTCIDPSCSGNGLCHLGKCVCYKGFKGDNCQLPDKLNLTHLCAR  DCSGHGQFDWDTGRCVCHRFFTGRDCEQEMCRLRCINGRCHNQRCVCDKGWGGVLCDTRN  CDPRCDGVKGQCDKGTCICRTGWNGKHCTIDGCPNGCSNHGNCRRYGNSGYKCDCHAGWK  GSGCNIAMEMMCSNNVDDDNDGLSDCLDPDCCSSAACTRSPFCQTVQDPAEILLQKPKPS  STASFFKKMKFLIDNNSIQKETSKNAFNESQVSVIRGHVETRDGTPLVGVRVNVRIQPLY  GHTLTRNDGRFDILVNGGGSVTLEFTRQPFQSHTISVSVPWNQIITMETVIMDLQSGDFT  EPDPSLCGVGHDHHSMKPIVLSTWQHTQLGACPEKSTLIPESQVLQESVEVPGTNVHLVY  HSSETEGYKSVILIQMTPDTIPTNLALVHLKVYVQGVETVKVFEADPGLKYTFSWDRINA  YRQKVYGIVPVKVHVGYEYKGCTYVFWEVRSTTMTGFDLTSSEIGGWNLDIHHTYNFQEG  ILHKGDGSNIYLKEKPQELVCILGNGIQRKLDCAMCNGDASNNQVRAPVALASGSDGSLY  IWDDNFIRKLSPGRTEIVSILKTDSVFHKTYMTVSPVNGKLYISDYMHHRVIQIATMGPVQNLE  QNFKVIAGNGEECSTGLLDECGDGGLAIQARLLGPKGIAINKEGVIYIADNLNIRQ  ISSTGIITTLIGSHNQLRTQEPMSCDHSRPANQVQLHWPTALAIDPLDDSLHILDKNVIL  KLTKDNYIVTIAGRPSNCPIRSIGSLLSGILSDEEEASGIAAEVRLVDAQSITFGPHGEI  HVVESDQHRINRVRVITSDGRIHHFAGSKSKCDCKSKTCLCYDAKETLAAQALFNSLTSI  TVTPDGIVHIADNGNLRVFSIMSKLPQPDTNNKYKVYSPDTKEMYIFNDHGQHQQTVDIM  TGQYMYNFTYNVNSFFSKLVSVTDDIKNMIELTRDSNLQVTQVISPGNQRSKVEMNNLHR  LQRFTSPNNNSLSFTYKGTTGLLESKYLSNGQSYFYNYNDKGRLMETRQPTGEITSLVTD  INTTGSIVRVNTDSSDVISMATYGSVQSVMHGAAETQVTYLPDGGVVVMYPTNMSITIES  GGHPVLSNQHRMHFKRKIIGPNQLVHKLEWRFYARRRYSPSCGRKTLQRLGSKMRINGVN  LLSVEYDRVNHTESILNKDSQNILWIMYDDSGLPVQFLPCSEHHAMNITYNQRGQITHWQ  YGEMWEDLNYNRDGLLLERSRSGTVQYRFNYRYGKSSPTDIVMPSGKQYYLEYNSLGELE  KIRTPDLGYHHFNHITSIGKQRYLYHIPGLSYPYSEEYDGNGKLLMLVYPSEQRRVAYRY  NSYSQPTMVLFDETQVELEYNEQILKLSQSTISSGPYSCVETYAYSGSLVSSYDVTFPKD  DKLITGSFSYTYDDNFRITKIDAAFGVHINGTSTSYAYDTNTGKLKTLGPLNLTFRTMYD  SETISDKYVTVTRSYDKYGRVENTKYRFDRDSVLTLKVGYDVYNRIHRWQRNVNGEEIKY  MYMYDKDSNIIEVFINGQSAWRFSYSNNGNINRVTEDGIPKDLEYDTGDRIIKSGNKQYK  FDEDGFMAKRHDQDLKFNSNGQLMYVAKTGKYRYFYFYDSFGKLVLMESNGGETMQYFYS  DVSNPNRITHTYNRTSLEVTEYIYEPSGSLIAMTRGGLVYYIACDPMGSPIAVMNKQGHI  IKSIVYDPLGRVENDTNPSFEFSFGFQGGLYNPVTELVIFSSRVYDTDNGRWLSPSYKNI  IHNIQKILEAPALLNNYRFQYLVNTHTKTSYPILSVTEWMSMLGYDIRSLAPDVSYTGEI  RPKKKDTDLSLLPTSSAFECTFLQDMDSLLTMSIVPKSKLSPLQTRTDVRFAALGSIFGD  GVTLSYRNGHVEVGVMEKTPDWSKQLALVLVNGSEILDLQYIINGKDVHYFVKPESSKAG  EDLKTLGIYNDEIRYENGLNVTVKRPTHRRTETDIKLHGKHSIINIRYGTSLELERQRVL  SHATTRAVNHAWRREKWILQNSLTSQYQWTSYEVNEILTHGSARGYEGRYRQSQTPTEYP  ELSDDCNSIKLQKTNR |
| SroTEN-X4_ID\|364840 | MQVQTGPAMDSNYNYRPKGQGQSQRKRCLKDSRMNSSQGSCSSDEEFHSDDNLRPYEEVK  VAHQDKKLNGLGLTPEEHVELTAMDQKVHMMCSKNNNKTPRFYTYREGSEGEESEQNLSV  STKHASLSVNHQGSQCSISSNEMDNMHSMRNEVVSRSDSDPDNQNHHINSPLGQNKFHHA  ATAPGGLPPCFNPPPPPPPIEDIPHQTTVPQRLNRCMGPSYRGTYSDADMCQCRGHFVDS  NTALLHNHHSIRGHYSDNENPYHRGHSCSDSEHYHQVRPCVYSGEESDFEPHYLEQTASG  NVFIPDGQPRFPRTVPRSGSVPMSRSGELVSPQPIRPQCSNTVPSHFLNGEHHNSERQAF  LPHGQCGYPANMTSPPRSHDCGYTKFGNTPHYMKKKLKRWSWKWAALILVIICVGLLAAT  TYFAAHLTFEKNEKKEEVPGALNHTVLKFTQTPFHLDGSRPTYTTIPPLTLWQTYFNQKD  SKFVKFDFTIPSSARLAIYGRRNFPPTIAQFDFYQVYDGSTIKDPSAQTMRDRYKRSLPD  KQTALVQMMEKGKWYITIYNDRDQPQEIGVSRTTIDSMGSSCERDCFGHGLCDNQGRCQC  FNGYRGPYCSELECPRLCNGQGEYRQGVCVCHEGWKGPECDTPANKCENPTCNNRGRCVD  GQCQCDKGFTGPHCGIVTCIDPSCSGNGLCHLGKCVCYKGFKGDNCQLPDKLNLTHLCAR  DCSGHGQFDWDTGRCVCHRFFTGRDCEQEMCRLRCINGRCHNQRCVCDKGWGGVLCDTRN  CDPRCDGVKGQCDKGTCICRTGWNGKHCTIDGCPNGCSNHGNCRRYGNSGYKCDCHAGWK  GSGCNIAMEMMCSNNVDDDNDGLSDCLDPDCCSSAACTRSPFCQTVQDPAEILLQKPKPS  STASFFKKMKFLIDNNSIQKETSKNAFNESQVSVIRGHVETRDGTPLVGVRVNVRIQPLY  GHTLTRNDGRFDILVNGGGSVTLEFTRQPFQSHTISVSVPWNQIITMETVIMDLQSGDFT  EPDPSLCGVGHDHHSMKPIVLSTWQHTQLGACPEKSTLIPESQVLQESVEVPGTNVHLVY  HSSETEGYKSVILIQMTPDTIPTNLALVHLKVYVQGVETVKVFEADPGLKYTFSWDRINA  YRQKVYGIVPVKVHVGYEYKGCTYVFWEVRSTTMTGFDLTSSEIGGWNLDIHHTYNFQEG  ILHKGDGSNIYLKEKPQELVCILGNGIQRKLDCAMCNGDASNNQVRAPVALASGSDGSLY  IWDDNFIRKLSPGRTEIVSILKTDSVFHKTYMTVSPVNGKLYISDYMHHRVIQIATMGPV  QNLEQNFKVIAGNGEECSTGLLDECGDGGLAIQARLLGPKGIAINKEGVIYIADNLNIRQ  ISSTGIITTLIGSHNQLRTQEPMSCDHSRPANQVQLHWPTALAIDPLDDSLHILDKNVIL  KLTKDNYIVTIAGRPSNCPIRSIGSLLSGILSDEEEASGIAAEVRLVDAQSITFGPHGEI  HVVESDQHRINRVRVITSDGRIHHFAGSKSKCDCKSKTCLCYDAKETLAAQALFNSLTSI  TVTPDGIVHIADNGNLRVFSIMSKLPQPDTNNKYKVYSPDTKEMYIFNDHGQHQQTVDIM  TGQYMYNFTYNVNSFFSKLVSVTDDIKNMIELTRDSNLQVTQVISPGNQRSKVEMNNLHR  LQRFTSPNNNSLSFTYKGTTGLLESKYLSNGQSYFYNYNDKGRLMETRQPTGEITSLVTD  INTTGSIVRVNTDSSDVISMATYGSVQSVMHVKISQGAAETQVTYLPDGGVVVMYPTNMS  ITIESGGHPVLSNQHRMHFKRKIIGPNQLVHKLEWRFYARRRYSPSCGRKTLQRLGSKMR  INGVNLLSVEYDRVNHTESILNKDSQNILWIMYDDSGLPVQFLPCSEHHAMNITYNQRGQ  ITHWQYGEMWEDLNYNRDGLLLERSRSGTVQYRFNYRYGKSSPTDIVMPSGKQYYLEYNS  LGELEKIRTPDLGYHHFNHITSIGKQRYLYHIPGLSYPYSEEYDGNGKLLMLVYPSEQRR  VAYRYNSYSQPTMVLFDETQVELEYNEQILKLSQSTISSGPYSCVETYAYSGSLVSSYDV  TFPKDDKLITGSFSYTYDDNFRITKIDAAFGVHINGTSTSYAYDTNTGKLKTLGPLNLTF  RTMYDSETISDKYVTVTRSYDKYGRVENTKYRFDRDSVLTLKVGYDVYNRIHRWQRNVNG  EEIKYMYMYDKDSNIIEVFINGQSAWRFSYSNNGNINRVTEDGIPKDLEYDTGDRIIKSG  NKQYKFDEDGFMAKRHDQDLKFNSNGQLMYVAKTGKYRYFYFYDSFGKLVLMESNGGETM  QYFYSDVSNPNRITHTYNRTSLEVTEYIYEPSGSLIAMTRGGLVYYIACDPMGSPIAVMN  KQGHIIKSIVYDPLGRVENDTNPSFEFSFGFQGGLYNPVTELVIFSSRVYDTDNGRWLSP  SYKNIIHNIQKILEAPALLNNYRFQYLVNTHTKTSYPILSVTEWMSMLGYDIRSLAPDVS  YTGEIRPKKKDTDLSLLPTSSAFECTFLQDMDSLLTMSIVPKSKLSPLQTRTDVRFAALG  SIFGDGVTLSYRNGHVEVGVMEKTPDWSKQLALVLVNGSEILDLQYIINGKDVHYFVKPE  SSKAGEDLKTLGIYNDEIRYENGLNVTVKRPTHRRTETDIKLHGKHSIINIRYGTSLELE  RQRVLSHATTRAVNHAWRREKWILQNSLTSQYQWTSYEVNEILTHGSARGYEGRYRQSQT  PTEYPELSDDCNSIKLQKTNR |

**Supplementary Table 2.** LC-MS/MS sequenced proteins of the TCAP pull down assay from the two wash stringencies.

| **Acetate buffer with 0.012M NaCl wash** | **Coverage (%)** | **#Peptides** | **Protein sequence** |
| --- | --- | --- | --- |
| glyceraldehyde-3-phosphate dehydrogenase | 3 | 1 | MTLKVGINGFGRIGRLVLRAALDKGLDVVAVNDPFIDLEYMVYMFRYDSTHGVFEGEIKTEGGKLVINGKAMSVYAERDPANIPWSKDQAEYIVDSTGCFTTLDKAGAHMKGGAKKVIISAPSADAPMFVCGVNEEKYTKDLKIVSNASCTTNCLAPLAKVIHEKFGIVEGLMTTVHAYTATQKVVDGPSNKDWRGGRGAAQNIIPSSTGAAKAVGKVIPDLNGKLTGMAFRVPVPDVSVVDLTCRLNKGASYNDIKAALKAASENELKGILGYTEDDVVSQDFRGDKRSSIVDAKAGIALNDNFVKLVSWYDNEFGYSYRVVDLIKHMYAVDSK |
| Extracellular superoxide dismutase | 18 | 3 | MIFLSKACNQCTSLRGARKMNCLLVFVVLVGAAFASKKNEANINIYLHLSDDAAQSDVDANYATTMHYAQCEMEPNPNQPASLHHHVHGSIEMSQLGDGEMTMSFHLTGFNVSDDFKDHNHGLQIHEYGDMEHGCDTIGELYHGEHVQGHANPGDLGDLHDDDHGNVTDTRKFDWLTIGHEDGILGRSLAILQGDHTSHTAIIACCVIGRSHAH |
| **TBS buffer wash** |  |  |  |
| Calponin | 2 | 2 | MAERMKPMGMDRALSSKMGAKYDPQAEAEVRQWFKQLLNEDIGEGAMTVEKNLKDGILLIKLLQKLYEETPNKPAACDKMKLKYNTSQAPFKQMENIELFLKGANAYGVPDNSLFQTVDLYEGRNMAMVIATILQVGTEAQRNGFNGPTCGSKPTERHQVQFSYEQLKQSHGVIGLQSGTNKFATQKGMRIGSVRHIADIRADDLTQDGQNHIGLQAGSNQFASQKGMTGFGAVRHISDIRADQYDKESAGIISLQSGTNKFDSQKGMRGGFGAVRHISDIRADQYSDDSKAHISLQSGTNQFASQKGMTGFGAVRHIADIRADDLDREAAAEVSLQYGTNRFDSQRGMTSFGAQRHVSDIKVTDLAEDMRRKMGYTPSQQEIQDMQDYSQQIHKIPRNVPYGPFCRRPETGNHYIEAQRNGFEGPTVGNKPVEKNARDFSYEQLKSSHGTIGLQAGTNKLDSQKGMTAMGAVRHISDIRADKFDKQSEGCITLQAGTNQWASQKGMTSIGAIRHISDIKVSKASQEGQADISLQSGTNKFDSQKGTRGFGAVRHISDIRADDLLREGTDKISPVISFTGGDSQKGMRGFGAQRHVSDIAVVDLADEFCKKTGYKPPKAVVKSDSKGDAQRNSFNGPTIGSKPTEKHVVHFTEEQKRAGHGIIGLQAGTNKCASQKGMKIGAARHIADIKADNLDRAGQGIIGAQAGTNQYASQKGMKIGGVRHIADIRADDMTQEGQGIVGAQAGTNKFANQSGMTFGAVRHIADIRADEASLEGQGVIGLQSGSNKGASQSGMSFGGRRHVSDIRVDDMSQDSQGITGGQSGTNQFASQKGMSFGAVRHIADIRADDATQEGQGVIGLQSGSNRGASQTGMSFGAVRHISDIRADEYSQEGAGTIGLQYGSNKGASQSGMNFGKGRGVADIPITDLAESMGYTQY |
| glyceraldehyde-3-phosphate dehydrogenase | 8 | 2 | MTLKVGINGFGRIGRLVLRAALDKGLDVVAVNDPFIDLEYMVYMFRYDSTHGVFEGEIKTEGGKLVINGKAMSVYAERDPANIPWSKDQAEYIVDSTGCFTTLDKAGAHMKGGAKKVIISAPSADAPMFVCGVNEEKYTKDLKIVSNASCTTNCLAPLAKVIHEKFGIVEGLMTTVHAYTATQKVVDGPSNKDWRGGRGAAQNIIPSSTGAAKAVGKVIPDLNGKLTGMAFRVPVPDVSVVDLTCRLNKGASYNDIKAALKAASENELKGILGYTEDDVVSQDFRGDKRSSIVDAKAGIALNDNFVKLVSWYDNEFGYSYRVVDLIKHMYAVDSK |
| protein maelstrom homolog | 1 | 1 | MCNDIEPSLRKEGRVFPNGIQDVVPIAHPRWKDYHKIPMENFENADSNYRGLWIQLENFVNPNGEKPEYPPFYCLGNDIKETAYCLEWIHGRACLGIPNRLKKVYELEGMVTDLYQHLGHSVSKTRVIEMITSNSWDYEPKTRCDYHEELECKYCSLGIIQRYSYAISDSFCGLLKIELTNRHLPVRTDNPVVALPPSSMKVKVPSQNQRFEKSPIKQSNSYSALAKKNFKDESSDESDDDNQSMTSLRRPHLPVNMAPAPRDPWAEQQKPKAANMGRGMWNGTPIPSKPPPPTATQTEFPSLGRGMGGVAGAPAVGFTSSAGDFPGLGRGSGLQGVGIGRGVPLSHVQQPAGQKFAGRAMAPDPSSAPVPPAAWVKEEPSKPTLNSISQSLQNIQAPRGPASTVPPGFKAMEPPVRQQQIPSRLIAPPQREVAGRGRGIMGTVDPSLKLPKGRGYAPPGMDVQKHLQMLQLARSSRQTN |
| tripartite motif-containing/E3 ubiquitin-protein ligase | 1 | 1 | MEFLNFIPVLQYCVEEIVTQKSKLCDSARVTSSDIINRAALLHDAIDKVKESLLLKTKRFLDSELKKMEFHEGELKTWISSLSSQNDVSNQNVVEVKDDSIGKIQNGDKKLSFTRLHQLCEIQHISLYFKSGEVVNESFVNSLFGSVQNFSITPPSPIRKNRRDTFPTIGVEIKTKFKCKGTAGNIHAIAPISENEAWICCGWGSKEMVLYNLDGEVLVSVMLDIPIDHIVSTRSGNVLVSSYNGNSIWCLDEQLTATKFATLSFVPRGMAVTDSNEIYVCGVERNRRHLIAKFAADGTLLSDITISPHDPHRIGVISEDKLCFSDYSKNNQRQLVIMDSSCNILHTYNGKNEPNIELENPFYPLGTQVDQYGNIIVADWNNDRVHLLDTTGNFSQFLVESGCGIERPCSLGLDRDGKLWVGNATGHVYVFSYCNWLT |
| Integrase recombinase xerD | 1 | 1 | MEDIGDAEVNPEMQRIISSENQILSSMQTMMDQNFKNFQTSMETTQKELSSSQLAKIEENIFGTYKFKRHGNEAQYRGNAQVITKLREADACLGSANITLDNVNKARTKIGEGIQLHTERQKLIKITDSSPLGWKVVAEYQANPIADDSDDEKKISRAITRAEKKEKQQRAARKPYDTSFRQHAFKKDETKSPTSKPGTCFRCGGKGHWAKDCRKKISDSEISILDTCLIRDSVRSDQEFHSNSLLLCSVTDQCSVQRVSPVGHLNSHLDRWVDTVRVSNERVDRTFALTKEILKKSDFSCMFNVRLIACLVGQIISMKAVLGNVVRIMTRYLYFCVDSRASWNSKILLSKNAIQEILFWSENIYKLNVNGAMLRTIDVSDLWEFELFSDASDTGYGGYIIPKGQLSDTGSPQQGGDGENVCGSWDTEESRRSSTWRELEAVHRMVHTHSDSLKGSSVAVYSDNKNVSQILEVGIRSKKENLQVIASDIFQLCDYNKITLYPKWIPRDKNQKADLLSRQTDRDDWGTRMWLVPPPVVISKTIKKCLSENVVGTLVVPKWISAPFWPILQSCTNISDSFVLPRNSIIVKDIGIREKVRADVAGSGAGSDFLIDLADKMSNNLLQEKSFNTIKTYHGSFRKWKIFAEKQGFQCLPAQPVHIALYITHMLDTGSSSHVVNHVIYSINWAHELHSFADPTNNSYIKCLQERQEASKRIATPTVSRKDPVSAEMLIRLCDLYLSSNDVMINRKDLIFSAVRKLAENNCLIDSEIKLSNEI |
| coiled-coil domain-containing protein 186-like isoform X1 | 1 | 1 | MSGIDPDEEANVTENPDPESESAALVNAENELPESNPNDGVEGEINVPVDNDHLPQENGSHPENVVDENVEENEAEEDGQGQDECVMNVEGDEKAEDEREADGGVIREGQISCGRDEQLIQGIGQEEAEADASLVRDISASESHTKYGASPHMPNENESEPINVLEQSKQRVNDAREEENISEGSNSVINLGSDGSNLESNLVQCGVNNVVENLSPENSEVVDGNAVIPEDEICCQNWTAEARPQLHVLSPTESIESYEPECDQEADISPSHEPEPANSDEPEPANSSDMNETCEQGGGDNEPEETLCVVVDPSSQNPALSDKESRTDHVTSSQYDTKTTSCSTESNKCLNFVNVNGSVTNHIESGNKISSYESDDDLLSELESELTNGPRTNQNSRNGPDAGVNPKLPNGMCGTIDHKVRQEIQDLQMQLKHSKDKLEQKEAEIKRLTIRGEEQETYVERILKERDSYLKEIRHLKSQDDLYLPQIKELEYTIVQQTNEIRTVKDKLASHDAAAKRTVAALQNELKVRVDQVTKMYEEANKEKDTMVVKYAMAEKKFIEGQKAIERLESKVRDLTKEKDGLTQRVKDIKGEKHRLIADLEAKAAEVQGLSKEVEKQSELISSSEVRIKWAQNKLRAELDAHKETKEELSKIRSKLKDAKDETEQIRRDCQAIIKTYQESEEIKSNKLDSELKIKESEFLQQQQEKTSQEEQYAATVKELASLKEKHQINLKEMESLKKKCQNLESQRDLNEQTMTKYKDMLQKQKKENKELQDRVEELIQVKSDFKRAQSTIKSLDAEISELKISNKDLQIDMEACRKREAEKLDLTKKLSEKNAGLQSENSVLHNKTVSQTEKIEKLTVDLQTLEIDFRDISERLKKEEVMRREETAKLKEKLDEKSKAVEELSAKVEDTKDEIKTIKRKHVNNVKDLTRQLQQAKKKLEHYETNGEGQKDSNSMGSRTSSSNSLNTMGVSENNPAQSNSYTNKYPGNSEPVQEYPVITEQVEPDSRVLIERIVKLQRGLARRNEKIEFMHEHIQQLVDEIQKKNKIIQNYALREESGTMTLERADLSKLDHRELSQVELSKKHSIMGSLYSSHQTDSAMTLDLSLEINHKLQAVLEDTLLKNITLKESLDTLGGEIARLSQENRQLQLQMQEKAKKR |
| fibrillin-1 isoform X4 | 2 | 2 | MSFDKSCGHKMRSFLLLPLVVAVVHCDTLYFRPRENPAVPGTPCQDGKSFCPDGSTCCVQESGEYGCCPLLNKVSLKLENVVCPDGQHQCKDGQTCCKLSSGQWGCCPLPKAVCCSDGKHCCPESTTCDTGTGKCKRGNQLTMDWFEKEPALLRSENVVCPDGQHECKTGQTCCKLSSGSYGCCPLPNAVCCSDGKHCCPSGTTCDVSSGKCKRGSSLVMDWFEKEPAKLRSENVVCPDGQHECKTGQTCCKLSSGGYGCCPIPNAVCCSDGKHCCPSGTTCDVSSGKCKRGSSLVSDWFEKEPALLRLENVVCPDGQSQCKTGQTCCKLASGQYGCCPLPKAVCCSDGKHCCPSGTTCEVSSGKCKRGSSLVMDWFEKQPALLRSENVVCPDGQSQCKTGQTCCKLASGQYGCCPLPKAVCCSDGKHCCPEGTTCDTGSGKCKRGNQLTMDWFEKEPAVLRSENVVCPDGQHECKTGQTCCKLASGSYGCCPVPNAVCCSDGKHCCPQNTKCEVSSGKCTRGDSLELQLIEQLLVTKPQGGLLSSVMCPDGASECKDGQTCCLLTSGKYGCCPIQDAVCCSDHIHCCPQGTRCDLQEQKCIGRGESLGIISMDMVQIKEGKSQESHSLIPLTANVECPDGQSECKTGQTCCKLSSGKYGCCPLPKAVCCADMKHCCPEGTTCEVSSGKCKRGDNILMDWFQKLPAVVKAESVMCPDGQHECKDGQTCCKLSSGQWGCCPLPNAVCCSDGKHCCPSGTTCDVSSGKCKRGSSLVMDWFEKEPALLRLENVVCPDGQSQCKTGQTCCKLASGQYGCCPLPNAVCCSDGKHCCPSGTTCEVSSGKCKRGSSLVTDWFEKEPALLRSENVVCPDGQSQCKTGQTCCKLASGQYGCCPLPNAVCCSDGKHCCPSGTTCEVSSGKCKRGSSLVMDWFEKQPAVIRSENVVCPDGQHECKTGQTCCKLSSGGYGCCPLPNAVCCSDGKHCCPSGTTCEVSSGKCKRGSSLVMDWVEKEPAVLRSENVVCPDGQSQCKTGQTCCKLASGQYGCCPLPNAVCCSDGKHCCPSGTTCEVSSGKCKRGSSLVMDWFEKEPAAVCCSDGKHCCPSGTTCEVSSGKCKRGSSLVMDWFEKQPALKILHTEIKPKNNVVCPDQTTSCPDKNTCCKNKEGKFGCCAYNNAVCCKSGTYCCPKGYICDTLPEICRMPEAKEAWKNTANIFIQNILKKRDYRSPHP |
| histone H2AX-like | 7 | 1 | MSGRGKGGKVKGKAKSRSSRAGLQFPVGRIHRLLRKGNYAERVGAGALVYLAAVLEYLAAEVLELAGNAARDNKKTRIIPRHLQLAIRNGEELNKLLSGVTIAQGGVLPNIQAGGNFAKKVSPAT |
| complement C1q-like protein 4 | 3 | 1 | MRKSNLILVAKFLSVSFIIQVNSFSLLTGNDSNGIENLGNTSSPREADILRQIVNQESLVRMSMVQSLQALMMDMLTCKTNYETLKKEVEDIRRENQKIIEKNSDLDRKVTNLSKTVFLGGFSFENFENGQQIIIEQLNVVKENHRELAGKINSSLGSVNEDLLSAKNHGVAFSAFMSSAKNYGQDQTWLFDSVLVNEGNHFNRASGTFTSPSAGTYVFSWATLTNPGNAAHPYLRINGVYKGKTAFNQLNSKEQLWSSGSNSIVLSLKEGDRVNIASGYLGAYAREEFSSFSGWKLF |
| digestive organ expansion factor homolog | 1 | 1 | MAPRKHGKGHLKRKSKSKHSQLSKRERKEIKEYGEIDPLNNGHGIGSRARFTKMADVEDHKLQELDSSDSSEEEVDAYQQLLASVHGKSQNIVGAESDEDEQEEEEVNGENDVSSGDDEMKEDGGEKEEQSMVENESSEVESESSGAESESEEKADNEGGDEIIEKEEDKEDYVVKESDPFSVHFETDLSEKFAEGLSSKENWESEEIKVKHKLAEGVENANRKLCNVDSLGNGAPLTPFQFSLFRVLNKYQDVYYPERSHKNGEEIRLVYCLHALNHVLKTRSRIITHNTRIKTKQQITGLEEYRDQGLTRPKVLIVLPFRDSALKVVNIMMKLLMSSDQSLVSNKKRFLTEYSEEDISEKKVPKPEDFEATFAGNIDDHFRIGLGVAKKTLKLYTKFYASDIVLASPLGLRTIIGVEGEKERDYDFLNSIELLILDQADIFLMQNWEHITNLMSHLHLQPQEAHGADFSRVRMWTLNGWSKYYRQTLIFSSLATPELNSLFNKHCCSILYHITIATNQRVFHKLPASSYSELADVRFDFFLKKILPTQKDAVMSQTLIFISSYFDYVRIRNHFVREDLEFAQINELKLRGVRHIIFYELPRYPHFYSEMCNMLQDTRRQTDKENMTCTVLYSGFDAHRLADIVGVDRASRMINSAKKTHMFVTGEDS |
| Galactosylceramide sulfotransferase | 1 | 1 | MLNKVDDGAMNVDEVEDVDLYITQLKDVFDSCDIYHRGYLSRSELLALCHKLQLEEQAEDIIDFEHFKENFVNILCQSTIERVEHENDVALSSSIHEQFMDADQFEDAEDEESDEILEEEGEENEEGVEKHEEEETKEEGDSPPDTPEFLDLQEEVTPKYVQGNKRYGRRSRPFQPEDFEEFLNTNAESEKVIPNQSTQPEKSTPEKDKVHKKPPLPPRKGTQHTESRNKVMVSTRFVVRKVIPVVFFLICVIVLTRRLPLDVRESTPPDSEDVIEDSVPDFETLHRPDERVGYWNKFVADNIKYQGERDNFVFIKCMKCATQTVAGALRRYAFTRRLNVVLPRDNNIYLGWPYLMDEVDYRPSEEKFNCLIEHAIYNRTIMEPLFPRNTPFITIIREPWSHFKSTFHYFKVDKIAGIEAEDPIEEYLNNIQKYEAIYKSHESPQYRYCVPDGFSVTKNLLSHCLGMPLGFPPGRVDYSQNEVLANEYIKKLDREFGLVMIMEYFDESMIFLKRLMSWSFKEIVYKKSNIGNYAKDAYPQYLKNIHTEWSSVDYLLYDHFREKMELKIQNAGFGFQSELKQFRQILSELNRFCASARRSSRINAKISFKHFTFTGYECAFLGEECMKCATETMGTIIRRFGLVRNLNFVVPVKNNIYLGWPFLIEELDYRPSKRPFNILMEHAIYNRTRMAKMMPNNTLYITIIREPWRRLTSSFSYFSLGYVVEPPVNLTTYVQNIHKYDEIYKQPEKKAWRFCFPNGFSPVQNLMAHCLGMPVGFPAGRKDISLNDTAIRQYIQELDTQFSLVMIADYFTESLILLRRLMCWTFKDIFYHSSNIGKHKLHDIVPTEEEFQIYFNFSRIDFLIYEHFNKSFWMKIQKQGPDFFDEVNQFKVVQLLIERFCFIENNSNSKGQFLIIPESKFNEELTISSEDCSFMTRYLLQDIRDQYNQVELGGDKSMFWYAKEPENGELPKRGCSFPLP |
| Fasciclin | 1 | 1 | MCSRKFCGQRHVGFLVCVLVCVIGFITSSSDVTRSKTIRQLISEINRLRGADDIILKNDLGSQYEQPDITAFVPSSEIFWRFKNVQSKYGLDLDNRESVTTLMLYHIGRGRVLSNEIIDGQTIISKHPSNYNLRFNIYHSGGQKASLIKVHIFTVNGAELLVRDIVGSNGVLYIIDRILAPVSSAKTLHDYLLYPDLPGYQFQSIARASIIDPDMKAKTNHIEHQFTSFVPPDAVLFPMPSYAQDILFFNTTLLKHTIHAHIVEDEIVFIPARGELKDLQSKRGTIHFTREGEDVYIQNNRIRARIVLPNIPVANGVVHVIDHILYFIYKTVFVRTNTTQALSIFASHLHGIPGDLLSHIQSTSETFTFFAPNNEAFAKIPRTFQQRLGQDDKLRSEVLGSHIVKGLDLSSSALTDGKTLKTINNFTLTIRKFNGDIYVQHDNIMAKIVQADIGCTNGIIHIMSSVLRLNQFTVLDAIKGNNQLLKISEMLKDFTVLEGILSGDTSLGGKVTVFMPSDLTIMSLRNDTRKNIFEKQPEKALKALKGHIIEGEALSSTEIYSTVLKYTFGGQRVEITNVDNEFTVEGSHIMAKVVTQDIWCSNGVLHIIDNILHIPTRNIMDELARHGDVSSISNILRLEGMKELSHALTTADSHFTMFVPINTAFSAIPRSRADTLFTNSTLFQNVLKAHVTSTGSKYTSDLYDGISMKAEEEVLHITRVSTDVFITNNNVRTRIIRPDIPAINGIIHVVDTIMYYPFYVAAEVMYNDPKLRIFYDLMKNLSDFSNLLQDEYSRMTVFAPSSKYLSSLSSNDLQRMASNPHVLRKIFKGHVIPNGLLDSKFIREHMQHKFTFRSTYGIPFTFEKQSTVEGTSIDAGFSNLRYDLDINRDGVACSNGVIYIIDGFLDYSFKNIIDEMKAQDKLKASLQNIMGIFPPGVEEDFRDTNNEFTVFMPASEAFLYLSQPEISYLYNLVNTSEKYELLERHTVNGSALSIERIRATCSDDNRTDCVLKVNVIFKEEDTQEIMLEWNGVQSKIIQSNILANNGIIHIIDRILFKVEEESTLPTNTLSTRGVNGATRNFSVLVSYFSLLTTVYLFYFLTR |
| monocarboxylate transporter 13-like | 1 | 1 | MTFKYEGTIKEHSRRKCDHDPVSEKDDAMALDPEVRQNPTDHGYAWVILAASMVATFIITGTFRAFGVLYVELIIMFHSNATMASLVQGILIFTSSTGFMFDLAAVLVLVFGLRRFTPRFWVVLGGHFYFFGFLTSSYAVSIEYLFFSYSCLVGLGMALSFSSIVVTMGNYFDKKRGFAMGLLMASGSLGSLVFAPFLRMLLDEYSVRGSLLIMAAITTHIIACGALLRPPAFYSKTSEKCRASVTQNAPREEKNSLIFNGNNICQINKPLYSSDPGIFLQNGKFRQFEHGTKSISTEMIHRSKDLDVQLTTPLGEYTIQNFCISVSAIDLNSEIKDKKVKINAINKEQFFNFKILKNPSFMRLLFAYTVGSIGTALPQSYLPALAIEQEAEATNAALLITVSSFFDLLGRLLIGYISDKRFVKRSYLIAVSMALSGVVQCLSPFYKDYWSLVLFSVFYGFFSNFMSALYSATLLDILGLDDFRSALSVMYSGYGIVSGAASPFIGDLRDKTGTYVTCFYLFGATHIISSIVLFTECISCS |
| TPR and ankyrin repeat-containing 1-like | 0 | 1 | MNPIGGLSSVDQMGQWAFFQLQHCKFIGEQSLRHGNYARAFAMYNTAVEYCNMCGPFLNVQEELSNIFSNRSLVLSKMGDFRLALTDAQECIGLNPHGAKGYLRAAAAYKGLNNYYEALNILIQGHNVTLPQGDQEIAISFLQELIVTITQLQRHQGDCDSLLAEHKELNLESLETDSQEKLLQGLASSHHWEGVSLLITGEHRGPRSSLDFSIPNVSAKYVSIACLFEDLSISDLKRYGMQLAVALLENGSPYQDIEEKFGTPILHVILIKTLETGYDGLIELFFRNYLDTQEKKDAVDRYGHSALHVIVRLKTCSDEERNNLLLLCIQHGCSALIFDKTEKLPIDYCDHTDKCFESLSRIFNDIETVRTHVLELEEKGNSEKDEKQYEQALLYYSRAIKLSTRCDMLHKDSAILYTSRCTVYAESNRLQEAMKDAELAIKIDNTYMKGHWRKSQLLRRNGQNSEAFAAAMEGVNISEIANKDKCELVMESVKSFQCLSDFEKNERYDAFSDVPVELWPNVLQRLSKEAEWLCIKQLVIGIGQDNELKGIARNTDFSTIRYGSLFTFIIQQQARKDVSSWIVPLIVHITTNPEGSNNLTSFKEWEGDTPLHAAARFSLITGNTSMLMCMDKLKVPLEMIDQNGNSPLHSVVKVPKPSQFAAFLEVVEKLLQMGVSPDLRDGMGKMAVDYVHKCEDSKIIQLLSKGNGDEDNSLQPLVQQKEHTVSGNKEKGDSGEDKTKLPNEDNQAAKVDLKLSLLASQDMKNEEHEHSSKILPKSSEKTEFKLHRNEGDRAFKAHKLKLALESYRKAKDHMKAASKNEIVDLFCKISDCFLDLRQHSMVIKEAEHFKTFIENSYQAKLRLGKSFACLQQNKVAFFHLIDAYHLSRSLTDVNMDDLLFEIASVYALCDEDEIDFFNVRDLERFCKCDWVKALYRLIQNDEWCAAENVYMDLSTWTEGYKYSFDLKILCNINKVQKHPWVIQFLMELLDNGSDFHTIIIEKGESFFHACVRLTLATVVRKREQNIQDSHGNTVLHLAVNQRTFHVHRGVQMQVVQRLLIADVNPLIWNKQKKTVSKCAPRSFPDLLDLIKICEVQKKAKIREKEEEDKQKSLMLRKQMEIDKKKHQEQLRIEKELRKEKENEAVSFKQCVLRCKAGLESAKEKLKSNELRLACYDLIYIFNATKHKSDMHKQFEDEGVQLIVNALGDKENPDIPEKLTRINQKIFLRIVHGLADKEHWKQMHIAVTEYRKYHSPSDLKDFAAEMSVEQVLNCKTFQNKEDLLLTVITNMLHSGASLLSDGKHAIETVVQNNHFKILDRLFDFKANPNHLSVEDGDTPIHAALYIALDRDKGNMSLLAKFLEMYQNNPEKYPMLDPKAVNLFGESLFHVVAKAKNNSTTLEVTIMLCEKGVNSNIKDQEGKLPVEYLKSDKDKRLQYFRLASKDTQLERDSNDKDDNDDQTKDQSCTRKKSKSEVQVHDVPLKKEKLMEEIRRMIEGLQDISMYFEEDLKKNRKRHDSISKEQECSSHNQIETKESLDSGIVLDNILNEETKLQIDPHFFESHEWEIECTHEVWSKLLNPAFPQHWKIDIIQKLQNLARGEFSFNCCRKIGITSVNAEIEIHESTPILWEIAVCFSSKLSAIDDNCRSVYTEIIRVWDIVENQEKKEEALRRISESILRGEECVVKKYVNCSKMLHQKFAKGKRIPRKYFECSLDTEGVQKLIFPASHVVTEYHILKFYSFTTDMALSALQNMDCKKDFPFKVTETEHSVININSRRPVLLLGRSGTGKTTCCLYRLWNRFDRHWRKEVEQKIYEGNHNEEIAEENIEYNNFQQMFITKNLVLCNEVQKCFRELRNASEIGSKIGPRDYLPLPAKFQDLQNDQSPVFTTSRRLLLMLDAFLPDPFFKRDSSGNILVDVPGWTNEDDSLSFLQQFAENSESENDDSSDEDGHPNTNNDAVKFLKKPGIRKEVNYSIFSNVVWPRINKCNEHYHPSLIWTEIISFIKGSYEALFTKLGYLSKEEYIEHGRKKAPNFTGERETIYEIFLKYKHYLKQHCLFDETDVVRNIFNRMGHFRKDEWKIQEIYVDETQDFTQAELYLLLNICQNPNDMFLTGDTAQSIMRGISFRFIDLRSLFYHAKKSQEPFTDIQIPEKIHQLTHNYRSHTGILWLASSILDILVEYFPESFDVLQKDQGLFQGPAPILLESCDVSDLAVILKGKKQSTSRIEFGAHQAVLVVNDEARDNLPDELQQGIVLTIYEAKGLEFDDILLYNFFKDSQAEKEWRIVTNFLEKIRIPETNKTTTGKENDSLYEIDYSVFEEEGRPRPLFFDPNRHKVLNSELKHLYTAVTRARVHVWIFDENEKKRAPMFEYFKARKLVQAVKDNESSDLFWNRHSAS |
| forkhead-associated domain-containing protein 1-like | 1 | 1 | MRAYLRGHDGSDHQVLPKLTTVGCEGCDVLIKTGGVDMQHAVIEYNDQENVYVLKDLNTAQGTYVNDCRIQNAAIRLAPGDQIKFGYMGTLYEFVVDSQQNVSCPPVHTQQTWNQPLTLLQEVTNEQSMLGQTQQMSYTAQQGPIFNTASTLPFLQTGTATFTIPSTVWTPSDRNTLPRPPISMRGRPLSAGSARRVQTVGGTPVPSPAPSMPQSMRNGWVNANSGRQQVNQNVDISKIQEKEQKIMQLNGEVGRLRDVEMESFRKDAYIQTLQQMVHDLKSKFAEQQPLIMGQDVNLTQKICQLENEVTTKEQELNAVREQLNSLQCQQQPSSSTTATILETDNKETNQMKIELERAKKERNITSGLVTQMQKDMANKDTTISKLTREIEMLRKEVRERDIQISSVSNKLSKSKETPLKSTEDRDAREKELISLRQKFKTAENKMQEQQELINTLKQELEKSKVSIFDERSQQKKLETQIDQLKSELSDVQRTERVVRIDLEQATKRLERFRNRVVQAAYSSGSAKAPEGEITDDQLVETLKKLVEERTEFDAKVKEMEKQLKLTDSSSNVFKRNIIKLRTDLEKSIENLKTNGFLVTSLKHEMDLLQSVTGDESILWMRDCLLQLLTHVSAWEKEIETSLQTCGININLSNDGPGKHIVSMHTKLEVVQKEKENLAAKLANTEQQFKSELEMKIQVMKEENENLVKDAIEKTRAEGEEKLNKAIEELKFVEAEKRENAIAGEQKKIEELQATIEQLRESLQEKQREFEERLQEANTTIQQIDEYKAIQLELEAKIQALETEKLELGATLSTEAQDRDKKYEQDIEAFKEQNKQHSVTICAMEERLIKLMKKNKDYQEEITVLKKTIQEMKSEMIQLKDKAATAAQTKPMPPPKPKVIVQKPSEDYVAMEQLVVVLRKENSDLKNTIQSRDDVILGLRRDLAGAHARLSDITGELSNNNNNNNNNNNNNNNNNNNNNNGLRQQMAKLSKIIDKQKDEINTLQKQLSEEKAISIKFQSNMDEKNRRIKELELDVERERLEQKKQLDILDQEGRITSELTALGAQCRGERHEQVISRQREALAELRHRCKNLEQSKPPLPTQDQALQQVIMLKKELAEIKANQALSENNELSSGLEREVSRARGMLGSVNAEADMERSAHRETMDALDASESSYITLLRAMASCLEMESVDGLRPIGHIPKDERDRLLVERENTCQILANRIKVLQERIARKEELLQGYERDLAKLRQAQELANRNSKQVDNLANDVKSRTEETQYLRESLSRTRDRLDQEKRLNKAIKQRKTFHLENERSHQQQVPASHHCKEEDIFGKNSARRKASKEALKRRNYEINTLKKELCSKEQALYDTENRMYTLEHSIGLEDKREQEIIES |
|  |  |  |  |
| cytoplasmic dynein 1 heavy chain 1-like |  |  | MRHESEEVGEGDGEAPAVYTFSVDVHYSSSKVSSLVLIKRAQVVEAEKSFPSQIRVMNFSEGSPYETLHAYVSNAVSPFFKSYIKETGKAERDGDKMAPTVEKKISELEMGLLHLQQNIDIPEINLPIHPVVSAVIKKCAEEGRKPKVEDFGDKVEDAQFLNALQSGVNRWIREIQKVTKLDRDPASGTALQEISFWLNLERALLRIQEKRESPEIVLTLDILKHGKRFHATVSFDTDTGLKQAMATVNDYNQLMKDFPLNDLLSATELDRIRVALSAIFIHLRKIRPTKYPIQRCLRLVEAISRDLSSQLLKVLGTRRLMHIPYDEFEKVMTACFEVFGTWDDEYEKLQGLLRDLVKKKREEHLRMVWRVNPAHKRLQARLDQMKKFRRQHEQLRQVIVRVLRPTTTTKPAQAGTPGQEEADAKVVKPLVDEVADANAIEEVNLAYENVKEVDGLDVSKEGSDAWEAAIKRYDERIDRVETRITAKLRDQLGTAKNANEMFRIFSRFNALFVRPHIRGAIREYQTQLIQRVKDDIEALHEKFKVQYIHSTACKMSKVRDLPHVSGRIVWSRQIEHQLNALLSHVEVVLGKGWENHVEGQQLKNDGESFRLKLNTQELFEEWQRRVQGRQLVVTGRIFSVQSFKSKVMGKGNVLKLCVNFQPEVIALSKEVRNLRWLGFRVPLAIVNKAHQANQLYPFAISLIESVKTYQRTLEKIEARITIVLLVAGLRKEVQNLIAEGVGLVWESYKLEPYVLRLADTENGFQEKVDDLVAIEEEIELEVKALETCAYSNAVFTEILGKIQKAVDNLSLHSYSNLPQWVAKLDQRVEAALAGRLEAGLKAWTQVLLGQDQSSSDNTMDTDEPQKQANKPGGDPHIKPMVHELRITNQLIYVNPPVEESRVNIMQELFGWEAVITSLPRIRHSRYQVGLDVESESESTYRNVLTKLPGGQIVLEDAYSAIQAVMKEFDKYVKVWLRYQSLWDLNPDVLYSRLGLKIKLWMNTLEEIKSSRKTFDTSETQKEIGPIIVQFGKVQSKVSLKYDSWHKEVLSKFGSLLGNEMTEFHSAISKSRSELEQQSIDTANTSEAVGLITQVQALKRRMKNWEKQVNMYKEGQRILERQRFQFPTNWLYSDNIEGEWGAFNDIMKRKDSSIQTQVASLQMKIVSEDKVVESKTVELLSDWDKDKPVTGELKPDEAVKALTIFEGKFNRLKEERENVAKAKEALELSEPGQLSASEERMQVAHEELQDLKGVWAELSKIWEQIDELKEKPWLSVQPRKIRQSLDGLLNQLKELPSRLRTYASYDFVKRTLQGYAKVNVLIVELKSEALKERHWKQLTKKLNVRWVLSDLTLGHVWDIDLIRHEHIVRDIILIAQGEMALEEFLKQVSEVWRSYELELINYQNKCRLIRGWDDLFTKVKEHINSVTAMKLSPYYKQFEEDALAWEDKLNRINALFDVWIDVQRRWVYLEGIFTGSADIKALLPVETSRFQGVSTEFLALMKKVTRSPLVMDVVNIPNVQKQLERLSDLLGKIQKALGEYLERERASFPRFYFVGDEDLLEIIGNSKNVARLQKHFKKMFAGVATITLNEDNSQVTGLLSKEGEEVVFATPVSIAANPKINEWLTLTEKEMRVTLAKLLQAAVKDVAKFKSGQIDANQYLEWIDKYQAQLVVLASQVSWSESVQSALSGIAASKNPADLTPLTDVLQVVDNTLNVLADSVLLDQPPVRRKKMEHLITELVHQRDVTRTLVKNKVNSPKCFDWLCQMRFYFDPKNPDVLQQLSIQMANAKFNYGFEYLGVQDKLVQTPLTDRCYLTMTQALEARLGGSPFGPAGTGKTESVKALGNQLGRFVLVFNCDENFDFQAMGRIFVGLCQVGAWGCFDEFNRLEERMLSAVSQQIQTIQEALKDMVGKKEKTSTAVELIGKQVRVNPDMAVFITMNPGYAGRSNLPDNLKKLFRSLAMTKPDRQLIAQVMLYSQGFRMAEKLAKKIVPFFKLCDEQLSPQSHYDFGLRALKSVLVSAGNLKRDKIQKVREGMLERGENVDERSIAENLPEQKILIQSVMETVVPKLVAEDIPLLYSLLSDVFPGVQYDPAEMTALRQEIKKVCQERYLTYGEQDEQGSQWVSKVLQLYQITNLHHGLMMVGPSGSGKSKAWQVLLKALERLEGTEGVAHVIDPKSISKEALYGTLDPNTREWSDGLFTHVLRKIIDNVRGELQKRQWIIFDGDVDPEWVENLNSVLDENKLLTLPNGERLAIPPNVRIMFEVQDLKFATLATVSRCGMVWFSEDVLSPEMIYENYLRTLKNVSLDEGEEESPKVLTGEAEETLSATMQVQTDCANILGPYFNSDGVVNRALEYAISLDHIMDFTRLRALSSLFSMINQGVRNILAYNNTHVDFPMQQDQVEKYITKYLIYSILWSMSGDGRLKSRQELGDFVRGITDIPLPPTTNQLIIDFEVSISGEWIPWQSKVPVVEVETHKVAAPDVVIPTIDTVRHEALLYTWLAEHKPLVLCGPPGSGKTMTLFSALRSLPDMEVVGLNFSSATTPELLLKTFDQYCEYRRTPNGVVMSPIQMNKWLVLFCDEINLPNMDRYNTQRVISFLRQMLEHGGFYRTSDHTWVKFERMQFVGACNPPTDPGRKPLSHRFLRHVPVVYVDYPGRISLTQIYGTFNRAMLRLVPSLKPYAEPLTNAMVEFYLMSQEKFTQDMQPHYVYSPREMTRWVRGICEALRPLDNLDVDGLVRIWAHEALRLFQDRLNNNNNNNNNNNNNNNVAFKNFPNINREAALVRPILFSNWLSKDYVPVDRERLREYVKARLKVFYEEELDVPLVLFDEVLDHVLRIDRIFRQPQGHVLLIGVSGSGKTTLSRFVAWMNGLSTYQIKVHNKYSAADFDEDLRNVLRRSGCKDEKIAFIMDESNVLDSGFLERMNTLLANAEVPGLFEGDEYTTLMTQCKEGAQREGLMLDSGEELYKWFTGQVMKNLHVVFTMNPSTEGLKDRASTSPALFNRCVLNWFGDWSNGALYQVGREFTNKIDLEKGNYMAPENIPAMHELPVSPSHREVVINAFVFVHLSLHQANARVLKRGGRITAVTPRHYLDFINHFAKLYNEKRSDLEEQQLHLNIGLQKIKETVEQVEELQKSLSVKRLELEEKNAAANAKLKQMVKDQQEAEAKKLMSQEIQTALMAQTKVIDEKKLDVKADLAQVEPAVIEAQQAVKSIKKQHLVEVKSLPNPPPVVKIAIESICTLLGETDLDWKSLRGIIMRENFISTIVHFTTDNISDDIRSKMKNKYLSNPDYNYEKVNRASLACGPMVKWAIAQISYADMLKKIDPLRNELKDLETQAKENLQKAEEVNKTIAGLEKSIAKYKEEYAMLISQAQAIKQDLSNVEAKVERSIALLKSLDDEKLRWESSSETFKSQMSTIVGDVLLSCAFMAYAGYFDQQMRRNLFTSWTTHLQTANVQFRSDLARVEYLSNADERVRWQANTLPADDLCTENAIMLKRFNRYPLIIDPSGQATEFLMNEYKEKGIKKTSFLDDAFRKNLESAVRFGNPLLVQDVESYDPILNPVLNRELRKTGGRILITLGDQEIDFFPKFTIFLSTRNPNADFPPDLCSRVTFVNFTVTRGSLQDQCLNQVLKSERPDVDAKRSDLLKLQGEFQLKLRHLEKSLLQALNASKGKILDDDSIISHLETLKKEASEVARKVEETDAIMAEVETTSQQYVPLATSCSSIYFSMEALQQVHFLYQYSLQLFLEIFHSVLQSNKLNGVKDYTARLNIITNELFQETYCRVGRGMLYEDRVTFAIQLSRICLKGMPSESTYEDEFIMFMRSQEALLGIGEKSAPEIPGLTSQQKSAMMRLTKHRAFKDLVQEIKGNEEFKAWIDSGTPEMDVVKVWNEDKPASPICKAVYGLLTIQAFRPDRTTSMARVFVEKVLGSSFLHNAEKELQLSGIVENEIKANTPVLMCSVPGYDASGWVDDLAAELGKQLTSIAIGSAEGFTQADKAINASSKSGRWVMLKNVHLAPNWLVSLEKKLHSLTPHPNFRLFLTMEINPKLPSNLLRAGRIFVFEPPPGVSANLSRTFTTVPASRMCKAPNERSRLYFLLAWLHAIVQERMRYTPLGWAKKYEFTESDLKVACDMLDTWIDSVAMGRTNLPPEKVPWDAIKTLLSQCIYGGKIDNDFDQRLLTTFIEKLFSPKSFEGDFTLVSNVDGHGKKILMPDGVQREQFVQFADMLENDSQTPSWLGLPNNAEKVLRSNLGSKLIAKLMKMQLIEEDDDLAYGATPEDEEKRLADGRPAWMRTLHTSVSTWMSLIPKAVGALKRTADNIKDPLFRFFEREVNLGAKLLHDIRQDLQDVMLICQGDKKPTNHHRSMMADLSRGIIPLSWRRYTVPQGITVIQWITDFSLRVKQLQAIVQTTQQGGTKELKNFSVWLGGLFIPEAYITATRQYVAQANSWSLEELYLDVKVLDNAGESMDACSFSVKGLKLQGAICKKNKLQLSTTISTELPVTLLRWIRTDDNAITAGKVTLPVYLNATRSHLLFTLDFDTEGEGSGKDHSFYERGVAIISSDLG |
| putative ATP-dependent RNA helicase TDRD9 | 1 | 1 | MTGLEQRNMTDLTPELGLDAIDDWFKIGKKSVQSLKTVPRSVTGGRYFDTITQRPRVAETENTFHLQRRTPAFSQTAYAEAYRQQEEEELMGYTHPHQQNRTSGINNDLEHLDLDSIATSTVAIPHDLVPSNAMDVYRNYDFEHKYESNLPISKHKDERIAGQQIILHRHLMFICFAKVIGTIESNQVTIVQGATGSGKTTQVPQYILDHYAKSGRYCNIIVTQPRRIAAISIARRVCSERKWQLGTVCGYQVGMDKQASEDTRILYVTTGVLLRKLVSKKNMLEYTHVVLDEVHERDQDTDFSLLIVRKLLRTNSRHVKVVLMSATFDCDLFAQYFALPVRDQLEPAPVVTVDETPHTVSEYYVEDFQGLGQIPPLDPYDATISKEAFALAARLIEEFDKMEVKSQGYVSRAGRVTKGRVYRMITRLFYEKFIPNYGIPEMQRCPLEKLVLQTKVFNMGEPKALLALALEPPNLDDIEKTILLLKELTFIGHVLADLPVDIKIGKLLIYGHVFGVLEECLIIGAAMSLKSLFSKPYKAHLDSYRHKLDWARGSQSDSLAILNAFKEYEARKNMGEFRRGVSEREWCKRHFLQQRRIREIAELVKELEQRLQQFNISKPGHRPNYKNHFSDDQERLLLKLVMCGAFYPNYFLKDPVDEESALREMSGHDPLNTVMVKGLPANQGLLYKEAVEDQFRNVGFNPEAFFEETKAYISFQWKPEYRGRVHPGVHMAVKLRQLRQSIAIDQLSSDEAHRLLQEIQRQQGETRGKLRSNRGDGSGDQRTHVSLPDTSVQYVSLLITNVVECGHFWAQYNENQNFEELNQVQGTLNSRNLSNVNAFSVGQLVAAPYTEQGDTQYYRARINSFDRQKRQERWIDVVMVFFVDYGNTETVDKAALRVCPQSITHIPYQAVECFLCELRPSVRKCPDGCWTREATDHFRTWVDQQSLYGQVYSTVRDTLRVDLIQSCGNGRQVSFRQELINLGFGERGEENYLSKKDHEQRQAPQTVRTQEQRTQPTEDWLGTAIRTSAPLSAAPLTRTRGQRIHLKGPHNPYEMQFYSLTNVGRLRAAKVDPDSVNSVAIDDEPESPHARMMIAGYVGLNAVGSTMIARDTTIMPLIPGLPALVSLLFAPIAEFRVDQRKENYIGTICGLGLDRFTGQPAMPDHDMEVAFDIPITLEDIFKINGVRMAINIVLGSEQAVSNWGEDAIYKLQDSARKKLMSVIQGTAREHYDAPATETPYRWDQIDPADILEHDLHNTPADSVVLLHLHKEKRRHAEWLRQAAESATSREPILCRLCNMTWQTPQLLQLHLETRRHKEKEDELEYCKSKKNV |
| Extracellular superoxide dismutase | 12 | 3 | MEKEEGLAESKIAHLEDEVKFLKSNLSALWEIVHEHDTLHGIVRPPKPYKRVVRETKKHKVHFHIHLDIPGSHPAHEEDCHNELEPVASVTKETKEHRVNMDFFMNIPDHPASDPVKSVERETEEHHVNMDFFLDIPYHPPPHHYSATSSDSDHEDCPVRRVVKETKHHVVHVDFFMDVPDHPAEHKVATTQTQEMHYAQCQMRPNPYQLASLHHHVHGSIEIAQRGEGDVTMSFHLTGFNVSDDFKDHNHGLQIHEYGDMEHGCDTIGELYHGEHVQGHANPGDLGDLHDDDQGTVTAKRSFDWLNIDEADGILGRSLACEMAPNPNQPASLHHHVHGSIEMSQMGDGDITMKFHLKGFNVSDDFKDHNHGLQIHEYGDMEHGCDTIGELYHGEHVQGHANPGDLGDLHDDDHGNVEDSRTFDWLTIGHEDGILGRSLAILQGDHTSHTSIIACCVIGHSHAH |
| cathepsin B | 17 | 4 | MKVLVLSLLVAGAMSALVEFRDRDIFEPLSDEMIWFVNKLNTSWKAGQNFDHIAKEDRLAHVKMMCGTYLNTPPELRLPEKKMEARKDLPDTFDARTQWTNCPTLKEVRDQGACGSCWAFGAVEAMSDRICIKSQGKSNAHISAEDLTACCRTCGNGCEGGFPSAAWSYYERDGLVTGGQYNTHQGCLPYTIKACDHHVKGKLQPCSKKIGPTPRCEKKCEAGYNVTYAKDKHYGMSAYSVHGVEKIQTEIMTNGPVEGAFTVYADFPQYKSGVYKHTTGQPLGGHAIKILGWGTENGDDYWLVANSWNPDWGDQGFFKILRGQDECGIESQISAGEPKLQ |
| carbonic anhydrase 2-like | 8 | 2 | MAPIQWILVFFSVFALSLDAAEWHYSGEHGIYEWPHEFEQCGEVRQSPIDFPKVEDMTYSKMTPIEFHGFDDPTKYRLKLENNGHTVKVSIESGDVYIEGGGLPGRFKTAQFHFHWGHSGDEGSEHTFDGHNFPMELHVVNYNEKYGNISNAATKDGDGLAVLGFWYQVSDHDNYALAPLINELSHVPVKGSKMNLTNVNLGLLLPIHMDEYNPHFYRYSGSLTTPPCFQSVVWTVFDNKMPISRAQLHMFQNLHEDKEVEGDHYLIDNYRPTQPLNGRTIYRNFEVTSTGHVEQQHQQAQQEVAKPKRKAKLNVRLHLAD |
| perlucin-like protein | 2 | 1 | MVMKAWLIFVCWLCVSAVFGCSEKQAFICSPPFEIYEGNCYKISTDRVSGDEAFARCSRAGAYLANFEILEEAMLMKQKLKKMNTGLHFYVGGRNINRRKPGGDWRWIRNGEMKEMTYFAFGISQPGGSDSDPQDCMFFYAGERYTFHDVGCDNGLHFYVGGRNINRRKPEGDWRWIRNGEMEEMTYYAFGAGEPNGNDGAPQDCMFFYAGERYAFHNVFCDNDSYLRGDVCEKMRTGLFVVFLLCVTVGLGCPDKQSYICTPPFQVYEGNCYHISRDKVSGDEAFARCSRMGAYLANFETLEEAMLMKQKLTEMKSGLHFYVGGRNINRRKPGGDWRWIRNGEMKEMTYFAFGSSQPDGSDSAPQDCLFFYAAERYTFHDVFCDHDSYLGGVRNTVVHYSGFGIRYIVQIYT |
| elongation factor 1 alpha | 2 | 1 | MGKEKVHINIVVIGHVDSGKSTSTGHLIYKCGGIDQRTIAKFEKEASEMGKGSFKYAWVLDKLKAERERGITIDIALWKFETNKYYITIIDAPGHRDFIKNMITGTSQADCAVLIIAAGTGEFEAGISPNGQTREHALLAFTLGVKQLIVGVNKMDSTEPPYSESRFKEIKGEVEKYIKKIGYNPKAVPFVPISGWHGDNMIEASEKMSWYSGWEIERKEGNAKGKTLMEALDAILPPKRPTDLALRLPLQDVYKIGGIGTVPVGRVETGILKPGMVVTFAPPNITTEVKSVEMHHESLPEALPGDNVGFNIKNVSVKEIRRGNVCGDSKNDPPKGAKNFLAQVIILNHPGEIKNGYAPVLDCHTAHIACKFVEIREKCDRRSGKVLEEAPKMIKSGDAAMVLMVPSKPMCVEPFSKYAPLGRFAVRDMRQTVAVGVIKEVEKQEAAAGKVTKAAQKAGGKK |
| X-box binding protein | 2 | 1 | MTILTWALLLVLVGTVQGCCPPPQLEGITVGGGVTVTKSIFNPYSGYSKIYHDEVKRKFASDVYITSKKGGSMTLFVLEDYAGGKRYMVLNGTCRVAPLKGDFNRELCVSPSNYKDKMVLGLGNETILMDLYSVNYSKSKPDFDITGDAMVFPLTEQRCVLQSEMIVAKKGSKFVARESKILYNTTARISDSSIFDIPKECQNNTQLKFEPHFHCYSSLHVKTMIKGKQISRAHCPSSAVSMHSACVLLLCLISACSAFNIRDTASCCSPSELQGIAYSTGIVFASGQEVSFVGNSWYYYSESEQKIATLSFILIPSLGNITVMVKTLQDYTTSRKYTVTNGRCNITSLSDPFARSCLPSSAVNI |
| Transforming growth factor-beta-induced protein ig-h3 | 1 | 1 | MCSRKFCGQRHVGFLVCVLVCVIGFITSSSDVTRSKTIRQLISEINRLRGADDIILKNDLGSQYEQPDITAFVPSSEIFWRFKNVQSKYGLDLDNRESVTTLMLYHIGRGRVLSNEIIDGQTIISKHPSNYNLRFNIYHSGGQKASLIKVHIFTVNGAELLVRDIVGSNGVLYIIDRILAPVSSAKTLHDYLLYPDLPGYQFQSIARASIIDPDMKAKTNHIEHQFTSFVPPDAVLFPMPSYAQDILFFNTTLLKHTIHAHIVEDEIVFIPARGELKDLQSKRGTIHFTREGEDVYIQNNRIRARIVLPNIPVANGVVHVIDHILYFIYKTVFVRTNTTQALSIFASHLHGIPGDLLSHIQSTSETFTFFAPNNEAFAKIPRTFQQRLGQDDKLRSEVLGSHIVKGLDLSSSALTDGKTLKTINNFTLTIRKFNGDIYVQHDNIMAKIVQADIGCTNGIIHIMSSVLRLNQFTVLDAIKGNNQLLKISEMLKDFTVLEGILSGDTSLGGKVTVFMPSDLTIMSLRNDTRKNIFEKQPEKALKALKGHIIEGEALSSTEIYSTVLKYTFGGQRVEITNVDNEFTVEGSHIMAKVVTQDIWCSNGVLHIIDNILHIPTRNIMDELARHGDVSSISNILRLEGMKELSHALTTADSHFTMFVPINTAFSAIPRSRADTLFTNSTLFQNVLKAHVTSTGSKYTSDLYDGISMKAEEEVLHITRVSTDVFITNNNVRTRIIRPDIPAINGIIHVVDTIMYYPFYVAAEVMYNDPKLRIFYDLMKNLSDFSNLLQDEYSRMTVFAPSSKYLSSLSSNDLQRMASNPHVLRKIFKGHVIPNGLLDSKFIREHMQHKFTFRSTYGIPFTFEKQSTVEGTSIDAGFSNLRYDLDINRDGVACSNGVIYIIDGFLDYSFKNIIDEMKAQDKLKASLQNIMGIFPPGVEEDFRDTNNEFTVFMPASEAFLYLSQPEISYLYNLVNTSEKYELLERHTVNGSALSIERIRATCSDDNRTDCVLKVNVIFKEEDTQEIMLEWNGVQSKIIQSNILANNGIIHIIDRILFKVEEESTLPTNTLSTRGVNGATRNFSVLVSYFSLLTTVYLFYFLTR |
| deleted in malignant brain tumors 1 protein-like | 0 | 1 | MAQEEWTPLAKKYCPLMAGSIDKTDTEPHDHGIIRALSSSYKPNKDVAGDPHCTVFVSRLNPKTDEETLESELSYFGGINKIRLVRDIVTGHSRCYAFVEFKEERSAVRAERDGHMMEIDGHEILVDFELERTLPGWFPRRMGGGFGGKKESVDRDLMTEKEKNTENAREREVGPGTEENPGTGTGKEVVLQTEGGEKGVDPNTEDIDYKISIEALNKFERLPAYLNYVQMHLVTFRRVQLSQRRSARMIIVEDATLSGALTILMLQATAKTSAEVRLTHGNNISHGIVEVWQENQWKILCAWPQFFARDALVICKQLGFSQGTVLPMAAYGRQMGNYSHPFKGCSDGEDRIQECQFDKNYTPNVCATQDVHYGSVSCYDKLEEGGIQLSPVHKDTKDSQPNSGKVEIFQQQVFGQVCSTYFDEEEANVTCRQLGFLGGVPMTHEVEDTLPFLLTGLHCHGNESKLSDCYQELQICYTDQRAGVLCYNSSGVGFSLAESKTSSGGIAVISVDGVEGTICGQGWSNLDAQVFCRQLGYVTGISYSGKSSELARDVYMSSVSCLGVENSVIACKGSGWRNVNRTHCNHENDAGVYCYKSVRLSYGYQRENATLGIVEVFQKYYWTTLCADRFDQTDADVVCRQLGYSSARILLPGLFGRSGRYVYTISINCTGNETDILQCEHETGYCSSDNYASLLCTHEDTNPQLIVNIEHSDNGRVMVSQFDVKGTVCRNGWDNMAAMVLCREHGFKGGVVYSSSDELTRYQPIWVGSVKCSGQEKRIVDCVFNYSISPECGYDRSSAGVLCYNATGMKIRLAGGFNANQGRVEISRDSVWGTICHNRWSTSDADVVCRQLHYASGTSYRDSYHGAGTGPILMDRVSCFGSEKSIFECANLGWNQSADACKDHSQDAGVYCMPWVKLSQHNYGTVLAWKKNNYGPVCADGFDENAARVVCNELQYSYSIKFCCSGLGPEPKDLDLAMSDVHCDGTESGLRQCKNEYINPSCSDKNYAEVYCTNTNPAQISVQVGQSNIVEVTYVYRTGLLSAADFTDKDASVICRENGFLAGKALRHLRPKHKDLFWLRNVSCLGNENKFDECPNAGRVGNLGRFDNGIAAAVACSNANEPPKTQLRLSYGSKLSEGHVQLNLNGVWGSLCAGNLTDAEAKVICNLSGYRDGVALPGGSFGQNNPRGTPLMDYLNCTRDEKRLIDCVVSLSSSQSPCPLYTMSAVKCYETVRLTGSSRVDYGRLELWNDDGWYSVCDENFDDKDATVVCKSLGFTLGKAQCCSALGSNGTVNPILFTNFSCTGNEVNPLSCTYGKTGRCRSGKYASVLCLQTSTEEDLTIYVESFYGPVIISRYGLPGYVCSKHFDNFDATMICKTGGYQTGYAVNVYKDFTFPVVLGNLTCNGANEIDRCTFSKFSDDHGCSDSDTVAGVICSNTDKLLFKLDSQIKTKGIPMLEVGSNTLYLDDTYFDEAAVNLFCLNAGFAGGSKLSQSISLPSVHNIVTDIRCSPNAPTITHCKGNWDPRKSKSVPLYEISCFHQARLKNGGTFYGAVELYSSDSWGTVCPESFGQTEADVVCTTLGYKRSLPICCSPFGPIPSRHLFTEFQCSGSEKNILECSHTFRLSGVQCRQNDLHLKINGGKYFGDVKVSYMGVDGRICRDGWDDNDARVVCRQNNYIRGLAYSHYVEEGKQGSGPYWISNVQCQGNEGSLHECPHSPVGSVTECKSLHYAGVFCFNDQGIYYRISGGDPSGRSGRAEIFFDDNWGTMCNTNWGEEEATALCRQMGFHGGDPIEGPYKVPGKGVVYKSSYQCTGESTSLTDCPHSGWEEVVEGECTDHRNDAGVFCYQNVRIYNSIGRSKSSGPVLLFRQNQWYLLCDDGFTDHAATLVCQELGFSYGYASCCSTDGPLSYPIWMNHTLDCSLGTHVEECLQTKTCARRSYASVTCTHLPRPNPNTSPKPDAPLPSKAEDNKTAVAAISAIAGFLLFVVIVIIVIKCRQSKKKETGSDAHGSFREGVLNKSRDGSIHAQNPLHDLYDSTDVRIQNDSVQFHNHGEDRSPALAFSNINYDTIHFSPEQADSDNDTSDDHIVEHKDTKPLGIKSKMEDSSYAAMRPNFSASDLHVYDNQQNKTYRNCVTIENAPEDRPMPHFTRSLSEFVKK |
| fucolectin-1-like | 6 | 1 | MKRQLKSQHSSLSGKKYFNAAYGKGVSQSSTLGKTYPALKAVNGELLDFTHSKYEKQPWILIDLGQEFYVFEVEIYNVDKKVQCCANRFHDADIKVGRSLNSMLTCGHFKGPGKDKERIIIRCKNIIVARYVKIQITSETNEYLQLAEVLVWATEM |
| DBF4-type zinc finger-containing protein 2 homolog | 4 | 1 | NFEIFNSETYLFTEGCPPKPPCKGSTNCRATLVYETIGSSVCAVRCDTKCDSRCPPLPKCPAIKEGCTTIYKYSHISADKVCPTRCELQCECPPQPTCPPPPSQDCVTINRNKIINNRECPWECLHQCCKTDVKCPKPDPTNVYCVSTYKQYKTINGKRCVTGCFCVPDPDYATFKRR |

**Supplementary Table 3.** Putative protease cleavage sites within SRO teneurin and enzymes leading to the detected ~22 kDa TCAP. The score represents the predicted cleavage probability (cut off value >0.8).


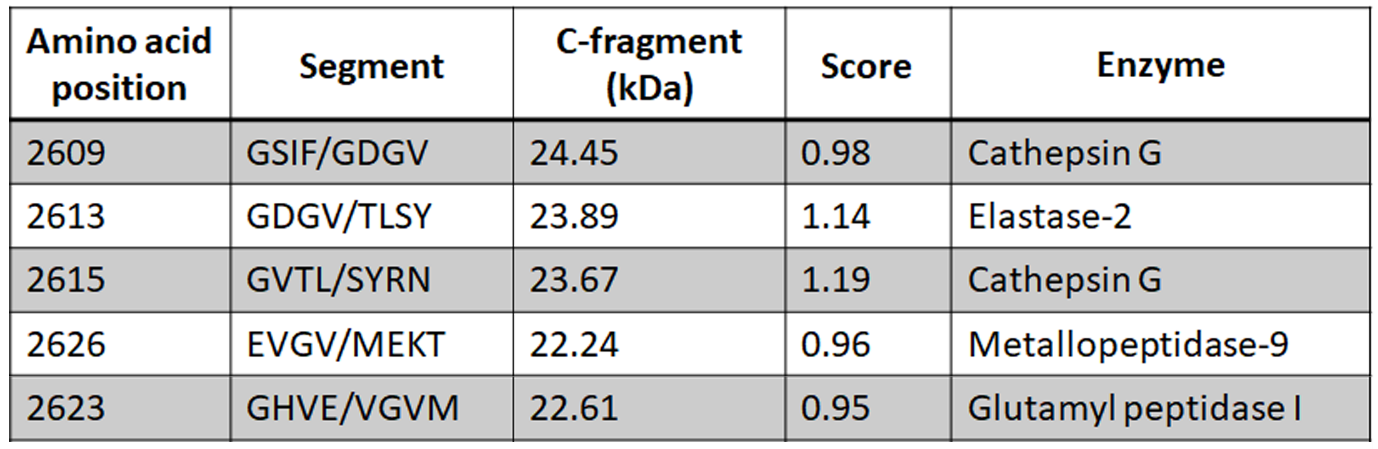


**Supplementary File 1.** ELISA showing serum titration of immunised rabbit after 1^st^ bleed against sroTCAP peptide.


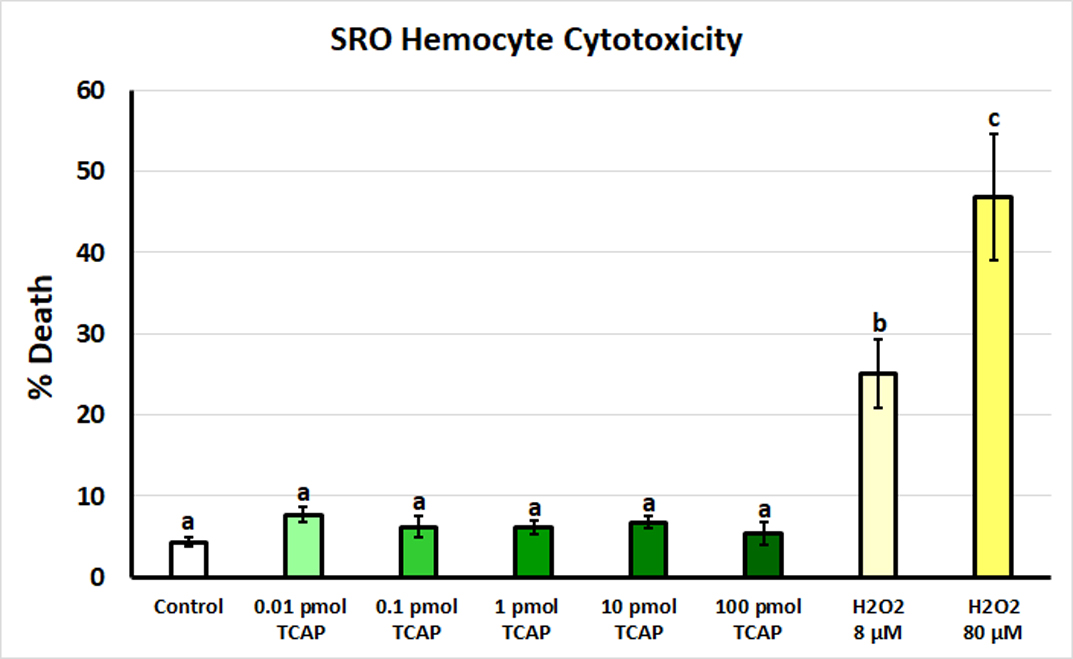


**Supplementary File 2.** Cytotoxicity assay using propidium iodide probe showing percent death of SRO hemocyte following 180 min of exposure to varying concentrations of sroTCAP. Hydrogen peroxide (H_2_O_2_) was used as a positive control.


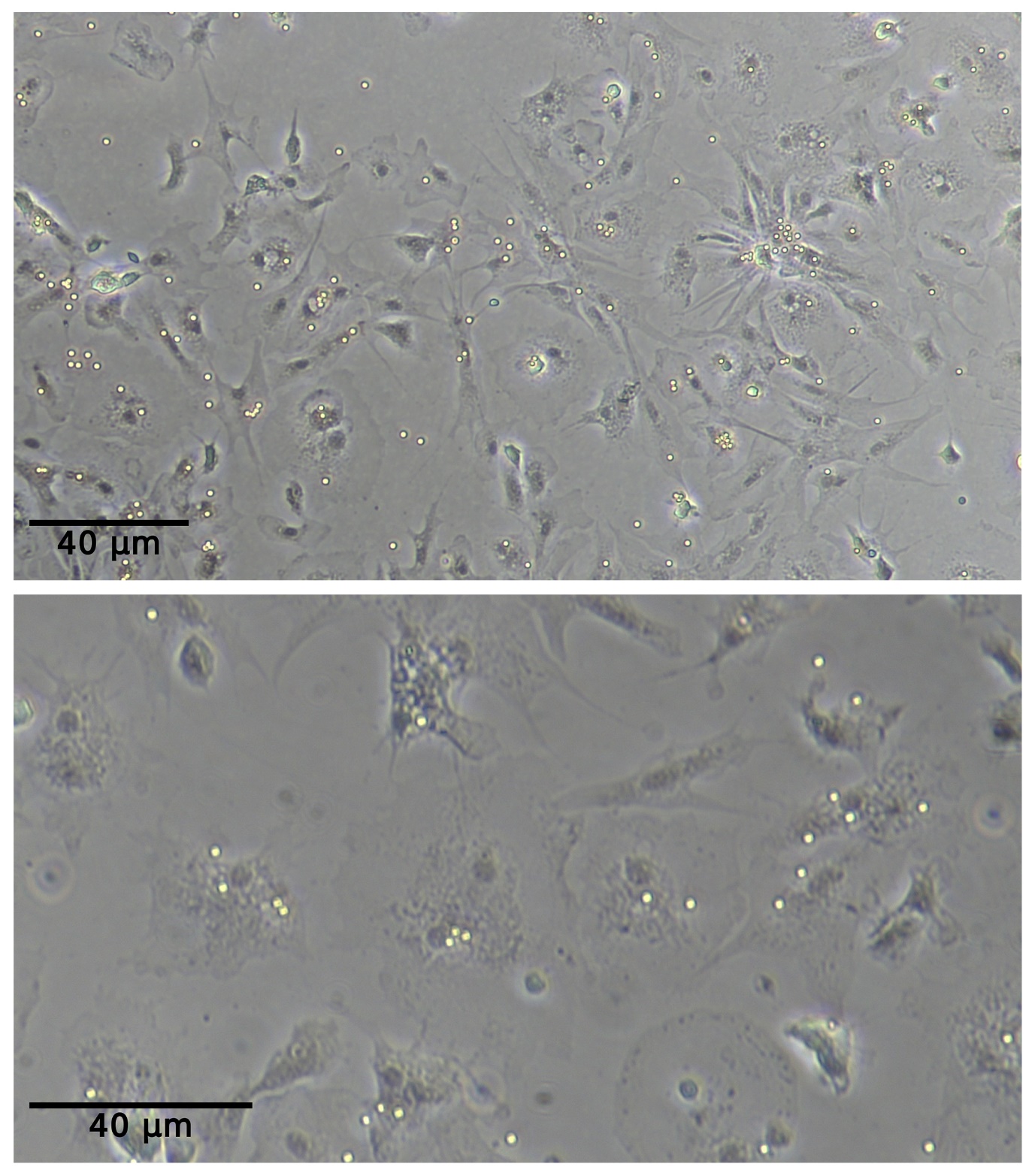


**Supplementary File 3.** Microscopy images of adhered *Saccostrea glomerata* hemocytes incubated with 2 µm fluorescent latex beads showing endocytosis as a measure of phagocytosis.


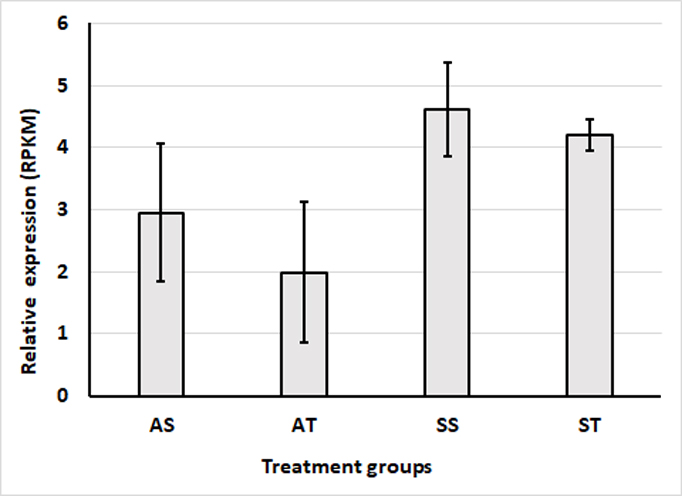


**Supplementary File 4.** Relative expression (RPKM) of *teneurin* in the 4 different treatments, showing average and standard error of the samples (n=3). Overall, teneurin expression increased under stress and slightly decreased following TCAP administration compared to the corresponding control. The differences are not statistically significant. AS- FSSW injection and exposed to ambient conditions, AT- stoTCAP injection (5 pmol) and exposed to ambient conditions, SS- FSSW injection exposed to stress, ST- sroTCAP injection (5 pmol) exposed to stress.
